# Influence of elongation and desaturation on chemosensory properties in acrylates and their corresponding 1-alken-3-ones

**DOI:** 10.1007/s00216-022-04332-9

**Published:** 2022-09-22

**Authors:** Patrick Bauer, Eva Ortner, Andrea Buettner

**Affiliations:** 1 Friedrich-Alexander-Universität Erlangen-Nürnberg (FAU), Department of Chemistry and Pharmacy, Chair for Aroma and Smell Research, Henkestraße 9, 91054 Erlangen, Germany; 2 Fraunhofer Institute for Process Engineering and Packaging (IVV), Giggenhauser Straße 35, 85354 Freising, Germany

**Keywords:** Butyl acrylate, 1-Octen-3-one, Homologue series, Odour threshold, Odour quality, Gas chromatography–olfactometry

## Abstract

Acrylates as well as 1-alken-3-ones are both known to be odour active substances but are generally identified in different materials. Nonetheless, butyl acrylate and 1-octen-3-one were both found to elicit a similar *mushroom-like* odour in previous studies. This led to the question of whether acrylates and enones with the same overall chain length generally elicited similar odours and whether they had similar odour thresholds. Overall, most of the investigated substances showed a *mushroom-like*, *geranium-like* or *fruity* odour. In contrast, short chained substances elicited *garlic-like*, *lighter gas-like* or *glue-like*, odour qualities, suggesting a correlation between the odour quality and the overall chain length. The results showed that only between the analogue structures butyl acrylate and 1-octen-3-one as well as hexyl acrylate and 1-decen-3-one could similar odour qualities be observed. All investigated substances showed low odour threshold values in air between 0.0032 ng/l_air_ (1-hexen-3-one) and 55 ng/l_air_ (1-dodecen-3-one). Overall, 1-alken-3-ones revealed a higher dependency on the chain length when compared to their respective acrylates. The introduction of a second terminal double bond led to a decrease of OT values in case of the acrylates and to an increase in case of the ketones that neither contained a second terminal double bond nor a double bond located close to the carbonyl group. Despite their structural similarities, the results suggest that both substance classes are perceived in a different manner and are therefore likely to be recognized by different types of receptors or are related to different activation patterns in multi-receptor stimulation processes.

**Graphical abstract:**

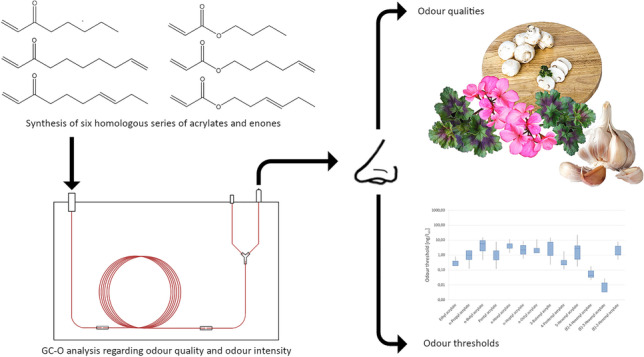

**Supplementary Information:**

The online version contains supplementary material available at 10.1007/s00216-022-04332-9.

## Introduction

Acrylates are esters of acrylic acid and tend to polymerize due to their reactive α,β-unsaturated carbonyl function. Because of their tuneable variability in physical properties that depend on the esterified alcohol, the formed polymers can be used in a wide range of applications such as coatings, adhesives, and the production of plastics or super absorbers as used, e.g. in diapers. Therefore, the overall global acrylate market is increasing at a compound annual growth rate of around 3% per annum and is extrapolated to reach a production volume of 5,686 thousand t/a by the end of 2023 [1]. However, their monomers are known to elicit an intense and unpleasant odour and could be identified as off-odours in acrylic paints (butyl acrylate, 2-ethylhexal acrylate) and adhesives (benzyl methacrylate, methyl methacrylate) [2, 3]. In these studies, butyl acrylate showed a *mushroom-like* and *geranium leaf-like* odour that influenced the scent of acrylic paints. It has been shown that most of the acrylates used for industrial purposes revealed a low odour threshold value (OT) with ethyl acrylate showing the lowest OT among the n-alkyl acrylate series [4].

*Mushroom-like* smell is, on the other hand, a characteristic trait of smell in the world of aromas. It is interesting to note that *mushroom-like* smells have been reported in different aromas as being important molecular constituents [5] and that *mushroom-like* smelling substances have been reported to belong to diverse substance classes [6]. As one of the most prominent aroma substance classes, some 1-alken-3-ones can be found in various types of food, being common products of lipid oxygenation processes [7–16]. This relates to their quasi-ubiquitous status and explains that 1-octen-3-one (*mushroom-like*, *metallic*) as oxidation product of unsaturated fatty acids is commonly found in different kinds of food and is a characterizing aroma compound with regard to the specific scent of mushrooms [13, 17]. Due to their low odour thresholds along with their high odour potency, small amounts of 1-alken-3-ones are sufficient to influence the smell of food, which can, on the other hand, impede the identification of 1-alken-3-ones in food. Especially with respect to complex aromas, the detection and quantification can turn out to be a real challenge. Since odour recognition by human assessors is nowadays still superior to instrumental detection, the method of choice is commonly gas chromatography–olfactometry involving human expert assessors performing the task using sniffing-ports in gas chromatography. Utilizing HRGC-O together with dedicated preparation of systematically designed molecular structures helps to reveal specific molecular features that give odorants their chemosensory meaning.

In this respect, there have been comprehensive studies achieved in our group, thereby systematically varying molecular structures and evaluating these with respect to smell. In case of the aforementioned alkenones, a series of novel findings could be established. Among others, it has been found that the introduction of a second double bond in these molecules further increases the odour activity in the case of the *cis*-isomers of the resulting 1,5-alkadien-3-ones, meaning that their smell was intensified, whereas a lower odour activity was observed for their corresponding *trans*-isomers [18]. Such observations might guide researchers not only to the revelation of the smell properties of such molecules, but also to their general biological meaning, or that of other compounds or processes that are associated with these. In this respect, one needs to keep in mind that smell and taste transport meaning, they communicate messages—be it about nutritional value, health, or potential threat. Common fatty acid oxidation products such as 1-octen-3-one, (*Z*)-1,5-octadien-3-one, and tr-4,5-epoxy-(*E*)-2-decenal are such potent carriers of information, to name but a few. On the other hand, even though few alkadien-3-ones have been identified in natural products (e.g. 1,7-octadien-3-one in essential oil of *Achillea teretifolia*) to date, their formation cannot be excluded [19]. Substances having not yet been reported in literature might simply have been overlooked in the analytical process. Chemical instability and potential rapid decomposition, together with concentrations below instrumental detection limits or chromatographic coelution effects, might hinder their detection and identification. Another reason may be that only few analytical data and standards are commercially available so that researchers are not referenced to such substances by the commonly established databases and identification and search algorithms.

On the other hand, there might be simply a limited cross-over of information between disciplines. While aroma research and odorant identification is very well established in the world of food analysis, this is actually not the same in the research area of material chemistry and other consumer goods. Even if smell has been, for example, commonly reported in paints and to some extent been taken as “normal”, there were no major endeavours to reveal the causative substances. Accordingly, they remained unknown until lately, with our discovery of the causative acrylates [2].

With regard to the unexpected smell effects, our previous studies revealed that butyl acrylate and 1-octen-3-one elicit highly comparable odour impressions to the panellists [4, 12, 13, 18]. However, we found that the odour threshold of 1-octen-3-one is up to 100 times lower than of its acrylic homologue butyl acrylate [4, 18]. Due to their apparent comparable molecular structure, one might postulate that both substances interact with the same or similar receptors; striking is their similarity regarding the α,β-unsaturated carbonyl function as well as the corresponding alkyl-moiety and, accordingly, coinciding chain length of both substances. However, their odour thresholds have previously not been compared. Reported odour thresholds in air partly depend on the methodology used for determination and can deviate to a large extent from one study to another. There are cases where differences in threshold values of up to a factor of about 40 can be found [18]. Such effects may arise, for example, for drawbacks und insufficiently controlled conditions in smell presentation or from variations in evaluation performance of the panellists [20–22]. Due to this reason, we consider it important to evaluate such odorants according to the exact same procedures in a comparative setup, as done in the present study in case of 1-octen-3-one and butyl acrylate.

Prompted by these two molecules, we further decided to expand this systematic analysis of odour quality and odour threshold onto additional homologues in the series of the 1-alken-3-ones and n-alkyl acrylates. Further aspects of this study were to additionally consider the introduction of a double bond. To the best of our knowledge, such systematic and comparative chemosensory information is currently not available. Therefore, the aim of this study was to provide analytical and sensory data on odour qualities and odour thresholds of 1-alken-3-ones and n-alkyl acrylates as well as their bis-unsaturated analogues (see Table 1). We deem it important to generate such data foundation for forthcoming investigations on food and non-food products, especially with respect to odour-potent substances that might otherwise elapse detection. All in all, we thereby complement our knowledge of structural features that elicit smell effects in humans.

**Table 1:** Retention indices on DB-FFAP and DB-5 capillaries, odour thresholds determined using GC-O in ng/l_air_ (median and range), and odour qualities of the investigated acrylates and 1-alken-3-ones

		Retention index^a^		Odour threshold [ng/l_air_]^b^				
No	Odorant	DB-FFAP	DB-5	Median	Range	Odour qualities^c^	Set^d^	Previously identified in^e^
1	Ethyl acrylate	1021	727	0.20	0.20–0.78	Geranium-like, lighter gas-like, garlic-like	1	Acrylic polymer resins [45]
2	Propyl acrylate	1092	800	0.98	0.12–2.0	Geranium-like, lighter gas-like, garlic-like	1	nr
3	Butyl acrylate	1175	908	5.7	0.47–15	Mushroom-like, geranium-like	1	Acrylic paints [2], figs [46]
4	Pentyl acrylate	1273	1003	1.9	0.12–7.7	Mushroom-like, glue-like	1	nr
5	Hexyl acrylate	1369	1103	2.9	1.4–11	geranium-like, mushroom-like, fruity	1	nr
6	Heptyl acrylate	1467	1203	2.3	0.56–8.9	Soapy, fruity	1	nr
7	Octyl acrylate	1571	1308	2.8	1.4–11	Geranium-like, fruity, glue-like	1	nr
8	3-Butenyl acrylate	1213	869	7.4	0.23–15	Geranium-like, green, carrot-like	2	nr
9	4-Pentenyl acrylate	1319	992	0.44	0.11–1.8	Mushroom-like	2	nr
10	5-Hexenyl acrylate	1423	1099	2.8	0.18–22	Geranium-like, mushroom-like, fruity	2, 3	nr
11	(*E*)-4-Hexenyl acrylate	1430	1095	0.044	0.022–0.18	Mushroom-like	3	nr
12	(*E*)-3-Hexenyl acrylate	1433	1082	0.014	0.0034–0.027	Mushroom-like, geranium-like, fruity	3	nr
13	(*E*)-2-Hexenyl acrylate	1407	1096	0.99	0.49–7.9	Geranium-like, mushroom-like, fruity	3	nr
14	1-Hexen-3-one	1113	774	0.0032	0.0016–0.10	Glue-like, geranium-like, fruity	4	Artichoke [11], honey [33], dill [9], butter oil [7], raspberry [15]
15	1-Hepten-3-one	1194	896	0.053	0.013–0.21	Geranium-like, mushroom-like	4	Green tea [47], shiitake mushrooms [48], blackberry juice [10]
16	1-Octen-3-one	1306	985	0.029	0.0036–0.23	Mushroom-like, metallic	4	Mushrooms [13], wine [49], olive oil [8], cocoa [12]
17	1-Nonen-3-one	1401	1079	0.030	0.0037–0.12	Mushroom-like	4	Tachibana orange [50], wine [16], yoghurt [14], raspberry [15]
18	1-Decen-3-one	1501	1183	0.72	0.18–12	Mushroom-like, geranium-like, fruity	4	nr
19	1-Undencen-3-one	1607	1283	1.91	0.48–245	Geranium-like, fruity, green	4	nr
20	1-Dodecen-3-one	1715	1383	55	14–220	Geranium-like, soapy, fruity	4	White bread [51]
21	1,7-Octadien-3-one	1344	977	0.92	0.057–1.8	Geranium-like, metallic	5	Essential oils from *Achillea teretifolia* [19]
22	1,8-Nonadien-3-one	1447	1175	0.12	0.015–7.5	Mushroom-like, geranium-like, green	5	nr
23	1,9-Decadien-3-one	1571	1176	0.23	0.11–3.7	Mushroom-like, geranium-like, citrus	5, 6	nr
24	(*E*)-1,8-Decadien-3-one	1579	1178	7.5	3.9–31	Mushroom-like, glue-like	6	nr
25	(*E*)-1,7-Decadien-3-one	1573	1175	5.6	2.8–22	Metallic, mushroom-like	6	nr
26	(*E*)-1,6-Decadien-3-one	1536	1173	0.37	0.012–0.74	Glue-like, fruity	6	nr

^a^Retention indices were determined as described by van Den Dool and Kratz (1963) [23]

^b^Odour thresholds were determined as described by Ullrich and Grosch [24]

^c^Odour qualities determined using GC-O analysis

^d^The substance is part of the named sample set as described in the discussion

^e^nr: compound was previously not reported as an odorant

## Material and Methods

### Chemicals

Acryloyl chloride, bis(cyclopentadienyl)titanium dichloride, 9-borabicyclo[3.3.1]nonane, 3-buten-3-ol, n-butyl acrylate, n-decan-1-ol, Dess-Martin periodinane, diethyl ether, diethylaluminium chloride, n-heptan-1-ol, 6-hepten-1-ol, 1,4-hexadiene, (*E*)-2-hexen-1-ol, (*E*)-3-hexen-1-ol, (*E*)-4-hexen-1-ol, 5-hexen-1-ol, 1-hexen-3-ol, 5-hexyn-1-ol, hydrogen peroxide solution, lithium diisopropylamide, magnesium sulfate, n-octan-1-ol, 1-octen-3-one, n-pentan-1-ol, 4-penten-1-ol, petroleum ether, phenoxyacetic acid, propionic acid, sodium bicarbonate, sodium thiosulfate pentahydrate, tetrahydrofuran, triethyl orthoacetate, trimethylamine, and vinylmagnesium bromide solution were purchased from Sigma-Aldrich (Steinheim, Germany). The educts 1-hepten-3-ol, 1-hexen-3-one, and 7-octen-1-ol were purchased from abcr (Karlsruhe, Germany) and chloroform, sodium hydroxide, and sulphuric acid from Carl Roth (Karlsruhe, Germany). Dichloromethane, ethyl acetate, n-hexyl acrylate, methanol, 1-nonen-3-one, n-propyl acrylate, silica gel (Normasil 60, 40–63 µm), and sodium chloride were purchased from VWR International GmbH (Darmstadt, Germany). All reactants were at least of reagent grade.

### Nuclear Magnetic Resonance (NMR) spectroscopy

^1^H-NMR spectra were recorded in CDCl_3_ using an Avance 360 (360 MHz; Bruker BioSpin, Rheinstetten, Germany) at room temperature at 360 or 600 MHz. Tetramethylsilane (TMS) was used as an internal standard (0.03 vol. %). Chemical shifts (δ) are quoted in parts per million (ppm) calibrated to TMS (1H). Coupling constants (J) were measured in hertz (Hz). The following abbreviations are used to describe multiplicities: s = singlet, d = doublet, t = triplet, q = quartet, m = multiplet. The identity of all intermediates and synthetic products was additionally determined by GC–MS in EI-mode as described in the following.

### Gas chromatography–olfactometry and gas chromatography-mass spectrometry

Gas chromatography–olfactometry (GC-O) analysis were performed on a Trace GC Ultra (Thermo Fischer Scientific GmbH, Dreieich, Germany) using a DB-FFAP (30 m × 0.32 mm fused silica capillary, 0.25 µm; J&W Scientific, Agilent, Santa Clara, CA) and a DB-5 (30 m × 0.32 mm fused silica capillary, 0.25 µm; J&W Scientific, Agilent, Santa Clara, CA) column. An aliquot of 2 µl of each sample was applied at 40 °C using cold on-column injection technique. The oven temperature was raised at a rate of 10 °C/min to 240 °C (FFAP) or 300 °C (DB-5), and the final temperatures were held for 5 min, respectively. Helium was used as carrier gas at a constant flow rate of 2.5 ml/min. At the end of the analytical column, the effluent was split in a 1:1 volume ratio and led to an FID and the odour detection port via deactivated uncoated capillaries.

Gas chromatography-mass spectrometry (GC–MS) analysis were performed at a 7890A GC-System (Agilent, Waldbronn, Germany) equipped with either a DB-5 or DB-FFAP column with specifications described above. Using cold on-column injection at 40 °C, a sample volume of 1 µl was applied to the system automatically and was separated using the temperature programs described above. The flow rate of the carrier gas helium was set to a constant flow of 1.0 ml/min. Detection was performed using a 5957C MSD (Agilent, Waldbronn, Germany). Mass spectra were generated in an m/z range of 35–300 using electron impact mode (EI-MS) at an ionization energy of 70 eV.

### Retention indices (RI)

Retention indices were determined as described previously by van Den Dool and Kratz using C_6_-C_26_ *n*-alkanes [23].

### Panellists

The panellists were trained volunteers from the Friedrich-Alexander-University Erlangen-Nürnberg (FAU, Erlangen, Germany). There was no report of illness at the time of examination, and the panellists’ olfactory function was fully audited. All panellists were trained in weekly sessions for at least half a year to orthonasally recognize more than 150 selected known odorants according to their odour qualities and name these according to an in-house developed odour language.

### Determination of odour threshold (OT) values

The odour threshold values in air were determined by GC-O analyses using a DB-FFAP column and (*E*)-2-decenal as an internal standard [24]. The commercially available and synthesised substances as well as the internal standard were mixed to obtain the stock solution with a flavour dilution factor (FD) of 1. This mixture was then further diluted 1:1 (v/v) to obtain the diluted mixtures FD2-FD16384. An aliquot of 2 µl of each dilution was analysed by each panellists until no odour could be perceived. The analyses were examined by five to seven assessors (five male, two female), with each experiment conducted once. Prior to OT determination, all synthesised substances were analysed separately by one panellist on both capillaries using GC-O regarding odour-active impurities to exclude potential interferences during the OT determination. The purity of the commercially available and synthesised substances was considered for the calculation of the concentrations in the stock solution (FD 1).

### Odour quality determination

Since some substances contained low concentrations of odour active impurities that could not be separated from our target substances during the preparation and were only detectable via their odour in GC-O analyses, GC-O analyses were used for the determination of odour qualities because impurities could influence the perception during odour quality determination in water. Therefore, the panellists were asked to describe the odour that they perceived during the GC-O analyses of the first FD and were related to odour qualities of commercially available reference compounds, such as (*Z*)-octa-1,5-dien-3-one (*geranium-like*), octanal (*citrus-like/fruity, soapy*), 1-octen-3-one (*mushroom-like, metallic*), and diallyl disulphide (*garlic-like*). Furthermore, the panellists were asked to record every change in odour quality in relation to degree of dilution. The panellists were free to choose odour descriptors according to the flavour-language developed in-house. An odour quality was chosen if more than 40% of the panellists named this specific attribute.

### Syntheses

Within this study, 18 substances were synthesised according to literature procedures and characterized in detail regarding their chemosensory properties for the first time. The purity and identity of all synthesised products were ensured by ^1^H-NMR and GC–MS. Detailed information about synthesis pathways can be found in the electronic supplementary information.

## Results and discussion

**Fig. 1:**
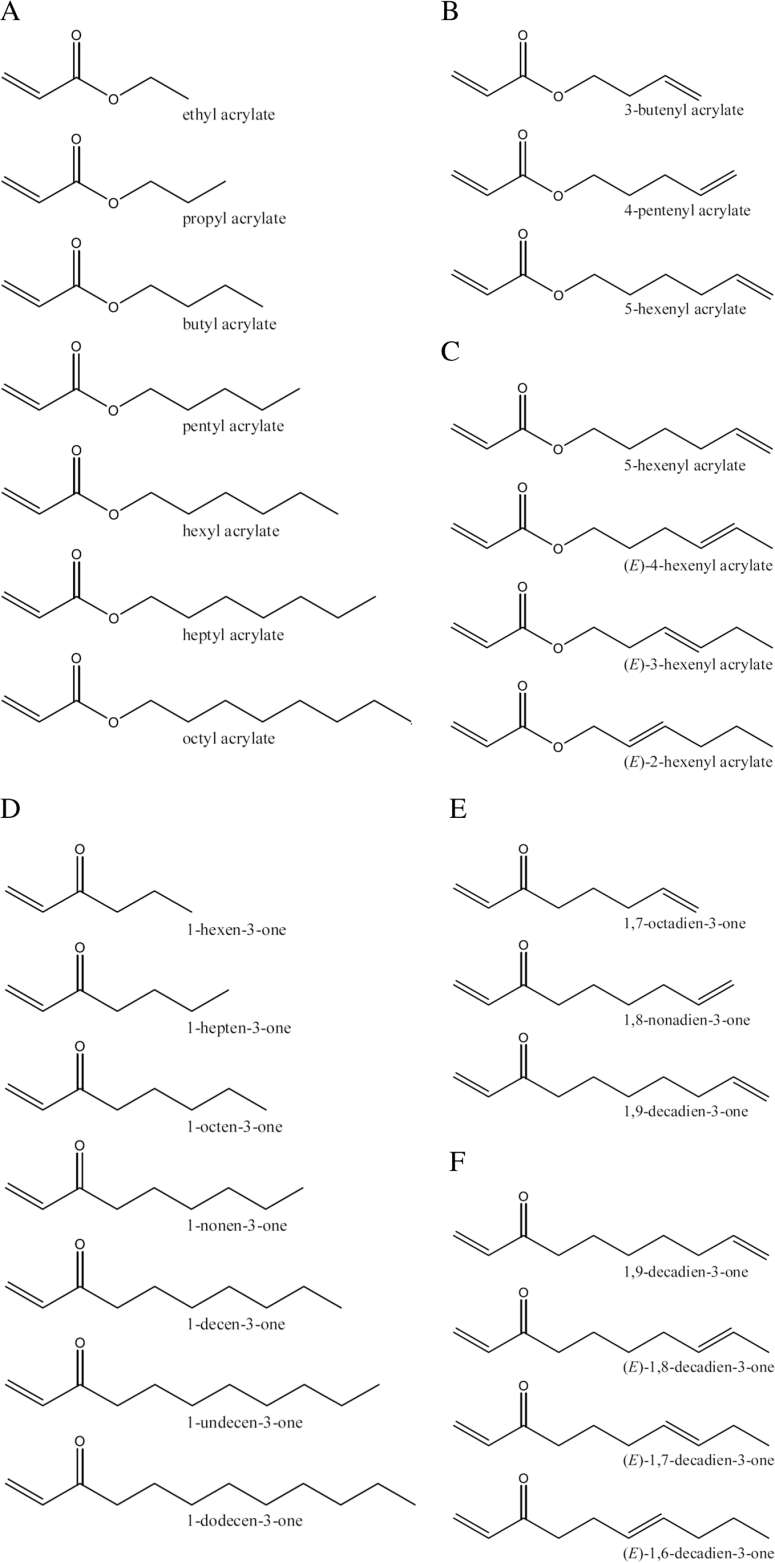
**A**–**F** show the analytes substances in their corresponding sets 1–6

Within this study, nine 1-alken-3-ones and nine acrylates were synthesised successfully, and their retention indices (RIs), as well as their mass and ^1^H-NMR spectra, were recorded to confirm their chemical identity. Thereby, the purity of all synthesised substances was above 95%. Further information regarding mass and NMR-spectra, as well as yield and purity, can be found in the supporting information. In Table 1, the retentions indices, the median odour threshold values, the OT ranges determined in air, and the odour qualities determined in air of all synthesised and commercially available substances are given. For the evaluation of the data, the investigated substances were separated into six sets as shown in Fig. 1 and Table 1. The investigated acrylates and the alken-3-ones were thereby separated into n-alkyl acrylates represented by set 1 (no. 1–7) and 1-alken-3-ones represented by set 4 (no. 14–20); substances with two terminal double bonds represented by sets 2 (no. 8–10) and 5 (no. 21–23), respectively; and sets 3 (no. 10–13) and 6 (no. 23–26) representing substances of the same chain length but with varying position of the double bond.

### Odour qualities in air

Overall, the investigated substances elicited *mushroom-like*, *geranium-like*, and *fruity* odours according to their odour qualities perceived in air. Divergent from this, short-chained acrylates and enones were predominantly described with unpleasant and pungent odour qualities such as *lighter gas-like*,* glue-like*, and *garlic-like*. The specific odour qualities of each analysed substance are given in Table 1.

#### Odour qualities of 1-alkyl acrylates and 1-alken-3-ones

Within set 1, short-chained acrylates, namely ethyl acrylate and propyl acrylate, showed a *geranium-like*, *lighter gas-like*, and *garlic-like* odour, which correlated well with the findings of a previous study in which such representatives have been described as “lighter gas-like, glue-like, garlic-like” and “lighter gas-like, sulphurous” [4]. Further elongation of the carbon chain initially brought about a *mushroom-like* and *geranium-like* odour and finally led to the *fruity* and *soapy* nuances. Since the three longest acrylates all showed a *fruity* odour, which was most pronounced in heptyl acrylate, it can be concluded that the *fruity* odour might be related to the overall chain length of the acrylates, namely their n-alkyl moiety. On the other hand, all n-acrylates except heptyl acrylate either showed a *geranium-like* or *mushroom-like* odour. As all n-alkyl acrylates have the acryl moiety as well as the ester group in common, it was concluded that the *geranium-like* and *mushroom-like* odours were associated with these groups.

Overall, the substances of set 4 were described with a *mushroom-like* or *geranium-like* odour whose intensity depended on the chain length of the respective 1-alken-3-one. As the shortest investigated ketone, 1-hexen-3-one showed a *glue-like*, *geranium-like* and *fruity* odour, which therefore differed from the other ketones as the perceived odour showed more resemblance to its acrylate analogue ethyl acrylate than to other 1-alken-3-ones. All other substances of the homologous series showed similar odour qualities as all of them were described to elicit a *mushroom-like* odour that was complemented with different nuances for C_7_ to C_10_. Further elongation resulted in a loss of the *mushroom-like* odour and the occurrence of *geranium-like*, *green*, *fruity*, and *soapy* odours in 1-undecen-3-one and 1-dodecen-3-one. Thus, the odour quality appears to depend on the overall chain length of the 1-alken-3-one. Similar findings were described in a previous study on the odour qualities and intensities of 1-alken-3-ones [18]. In this previous study, the panellists described 1-hexen-3-one as *metallic* and therefore also with a different, distinctive odour quality than substances of the same homologous series. With increasing chain length, the odour qualities were observed to initially shift towards *vegetable-like*, *metallic*, and *mushroom-like* odour qualities and finally to *herb*-, *citrus-like*, *soapy* odour qualities for 1-undecen-3-one and 1-dodecen-3-one. Since both panels consisted of different panellists and were trained to different in-house flavour languages, the odour qualities of both studies are not completely on par with each other. However, similar changes in odour qualities with increasing chain length were observed that indicate that it is rather a linguistic matter than a perceptual phenomenon.

When looking at the odour qualities of the investigated n-alkyl acrylates (set 1) and the 1-alkan-3-ones (set 4), it becomes apparent that the smell properties of the 1-alkan-3-ones are more influenced by the overall chain length than the respective acrylates, as the distinctive *geranium-like* odour of acrylates was perceivable in short-chained as well as long-chained homologues. Nevertheless, the chain length appeared to impact the smell correlation particularly with respect to the shortest compounds, which were both described with more unpleasant, pungent odour qualities, and longest compounds, which were described with *fruity* and *soapy* nuances. While longer and shorter substances differed in their odour between both sets, substances with a chain length from C_8_ to C_10_ showed similar odour qualities. This supports the hypothesis that these substances interact with the same types of receptors, thus leading to similar or the same odour perception. As these smell properties were not observed in all investigated substances, a general recognition of acrylates and 1-alken-3-ones by the same receptors is obviously unlikely.

#### Introduction of a second terminal double bond

Overall, the introduction of a second terminal double bond in acrylates (set 2) had a greater influence on the odour quality with decreasing chain length. While the same odour qualities were determined for hexyl acrylate and 5-hexenyl acrylate and only a loss of the *glue-like* nuance could be observed in 4-pentenyl acrylate, the perceived qualities of 3-butenyl acrylate shifted to a stronger *geranium-like* odour with *green* nuances that resembled of *carrots*. However, the fact that the *geranium-like* and *mushroom-like* odour remained predominant in set 2 also sustains the assumption that these odour qualities might be associated to the acrylic moiety as well as the ester group rather than the side chain.

As observed in acrylates, the introduction of a second terminal double bond in alken-3-ones (set 5) had a greater influence on the odour quality with decreasing chain length as well. While for 1,9-decadien-3-one only one nuance was described more specific by the panellists and 1,8-nonadien-3-one showed the additional nuances *geranium-like* and *green*, the main odour attribute of 1,7-octadien-3-one shifted from *mushroom-like* and *metallic* to a *geranium-like* and *metallic* odour. Nevertheless, the impression *mushroom-like* was named by 29% of the panellists and was therefore the odour quality with the third highest agreement among the panellists. Since the odour quality did not achieve the threshold of 40% agreement, it was not identified as one of the main odour qualities.

The introduction of a second terminal double bond had a greater influence on odour qualities of substances with a lower chain length. Since it was suggested that the acrylic moiety, the carbonyl-group, or the ester-group might be associated with particular odour qualities, an additional double bond might play a greater role with regard to the interactions between these molecular features and the receptors in shorter molecules due to their spatial proximity to these features. Corresponding analogues of set 2 and set 5 were described, in principle, with similar odour qualities but different tonalities, so that both analogues could be, in general, distinguished by their odour. This supports the assumption that both substance classes activate at least partially divergent sets of receptors.

#### Influence of shifting double bonds on the odour qualities

The results for set 3 showed that the introduction of the double bond did not influence the odour quality if the double bond was either terminal (5-hexenyl acrylate) or the closest to the ester-group ((*E*)-2-hexenyl acrylate), as both substances showed the same odour qualities as hexyl acrylate. However, the introduction of a double bond between these positions caused the main odour quality to change from *geranium-like* to *mushroom-like* in (*E*)-4-hexenyl acrylate and (*E*)-3-hexenyl acrylate and brought about the absence of further tonalities in (*E*)-4-hexenyl acrylate. It is noteworthy that within set 3, only (*E*)-4-hexenyl acrylate and (*E*)-3-hexenyl acrylate revealed a mushroom-like odour as their most pronounced odour quality, while both of them also revealed lower odour threshold values. The simultaneous occurrence of low odour thresholds and a food-related odour quality appears plausible in the context of evolutionary demand to find and evaluate food using the human sense of smell.

The position of the second double bond in the series of the decadien-3-ones (set 6) showed a greater influence on the odour quality. The odour qualities described for 1,9-decadien-3-one (*mushroom-like, geranium-like*, *citrus-like*) changed marginally from a *fruity* to a *citrus* tonality when a second terminal double bond was introduced. However, the shift of the double bond into the direction of the carbonyl function led to a *mushroom-like* and *glue-like* odour in (*E*)-1,8-decadien-3-one and a *metallic* and *mushroom-like* odour in (*E*)-1,7-decadien-3-one. In both substances, no *fruity* or *citrus* tonalities were perceived by the panellists. However, the *fruity* odour re-appeared, when the double bond was introduced yet closer to the carbonyl function as in case of (*E*)-1,6-decadien-3-one. In this case, the *mushroom-like* or *geranium-like* odour was replaced by a *glue-like* impression. Accordingly, the position of the double bond has an influence on the odour quality of decadien-3-ones. Interestingly, the absence of the odour qualities *fruity* and *citrus-like* correlated with a significant increase of the odour threshold.

Overall, substances of set 6 showed a higher dependency on the position of the second double bond regarding their odour qualities. We conclude, accordingly, that in set 3, the odour qualities were mainly influenced by the acryl as well as the ester group rather than the second double bond. Due to the additional oxygen atom inside the carbon chain, acrylates might interact with odour receptors in different ways, so that the double bond and its position appear to be of less relevance with regard to odour quality. However, as this oxygen moiety is missing in the decadien-3-ones, the position of the second double bond within the chain might gain greater impact on the odour quality. Future studies involving larger cohorts might substantiate this assumption. It also needs to be kept in mind that for the sake of targeted investigation, the 40% threshold rule was incorporated in this study. Future investigations would need to be designed in a way that would allow for broader testing and more comprehensive acquisition of descriptors in larger panels. However, this necessitates the appropriate tools to be developed to allow appropriate and defined presentation of substances for large-scale olfactory testing. Such tools that cope with the challenges of smell presentation without matrix or device interference, substance stability and precise dosing, and the respective data acquisition are not yet at hand [30, 31]. Only then will we be able to comprehensively gain both the sensory descriptors and to correlate with the respective interindividual expression patterns of odour receptors. Moreover, one needs to keep in mind that other factors such as the so-called perireceptor events, namely metabolic conversion by enzymatic activity in the nasal mucus prior to receptor activation, might strongly impact smell perception [32].

### Odour thresholds in air

**Fig. 2:**
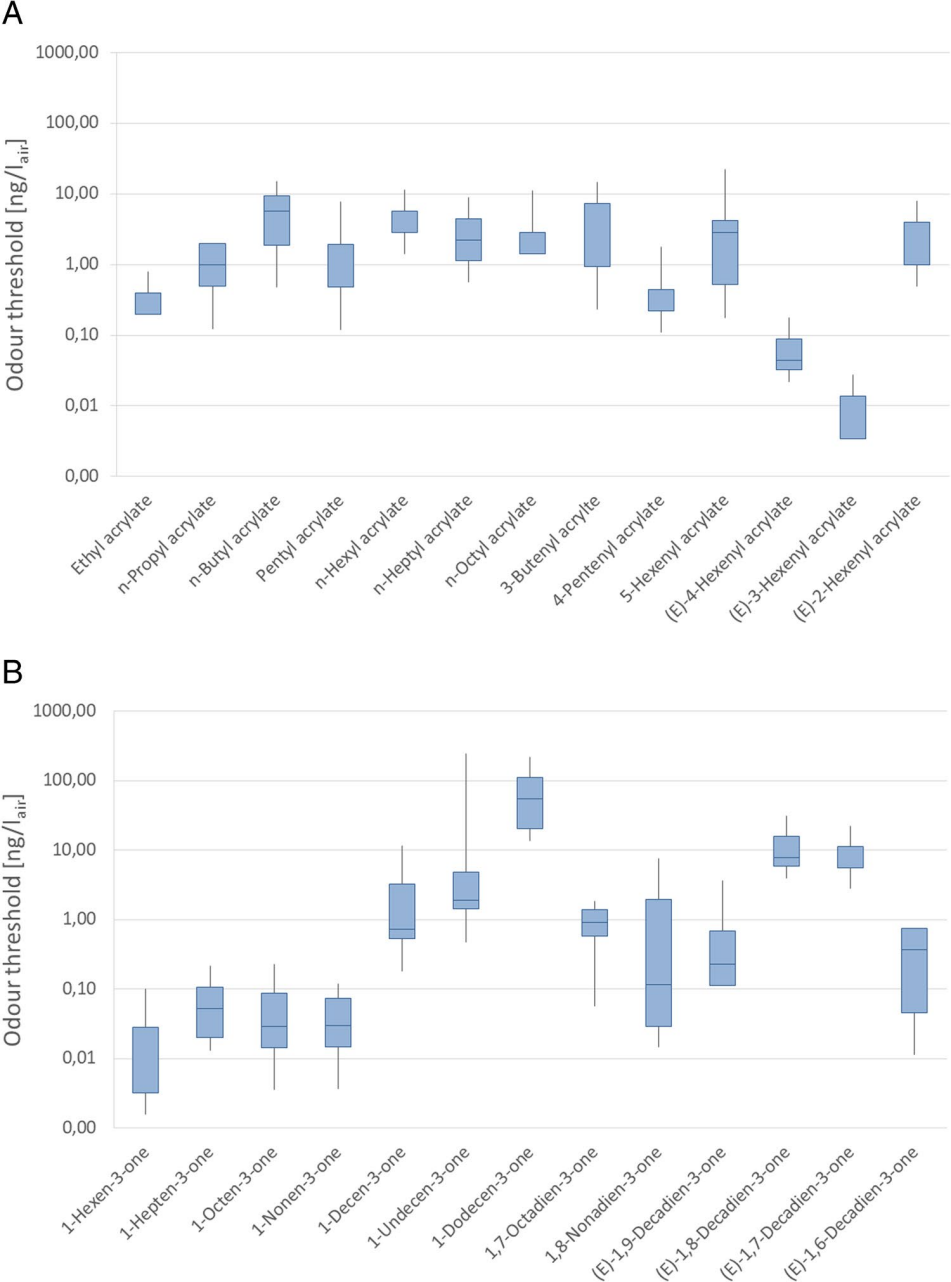
Box plots comparing the odour thresholds of all analysed substances

Containing either one or two terminal double bonds, the investigated substances tend to polymerize, making it necessary to investigate the substances as close to their time of synthesis as possible to avoid degradation along with declining concentrations. Thus, the mixtures had to be prepared freshly and had to be analysed within 1 week by as many accessible trained panellists as possible. The odour threshold values in air were therefore determined by either five or seven panellists via GC-O analysis. In general, odour threshold were found to be comparatively low as the determined OT values in air were between 0.0032 and 55 ng/l_air_ revealing the high odour potency of the majority of the investigated substances. The odour thresholds of the acrylates and ketones are compared in the box plots shown in Fig. 2. The findings for 1-alken-3-one and n-alkyl acrylates correlate well with the OT values found in previous studies [4, 18].

Odour sensitivity of panellists being either far above or far below average led to OT values that varied by a factor of above 500 between all panellists in two cases. Therefore, median odour threshold values were regarded to evaluate the obtained OT. However, OT values obtained using the geometric mean (data shown in the supplementary information) showed the same tendencies that were observed based on the median OT values. Since the median OT values appeared to be more representative in this case, median OT values are further discussed here.

#### Odour threshold values of alkyl acrylates and 1-alken-3-ones

Overall, the median OT values of the homologous series of saturated n-alkyl acrylates (set 1) fell within a range of 0.20–5.7 ng/l_air_. Unlike in other homologous series [18], the effect of the chain length of the esterified alcohol did not exert a pronounced effect on the odour threshold values of the n-alkyl acrylates. Hereby, the OT values increased with increasing chain length until hexyl acrylate (2.9 ng/l_air_) where they then remained constant between 2.3 and 2.9 ng/l_air_ when further increasing the chain length. The findings correspond with the propositions of an earlier study suggesting that the odour threshold of acrylates depends on functional groups of the side chain more than on the chain length itself [4]. Ethyl acrylate showed an OT value of 0.20 ng/l_air_ and therefore revealed the lowest median OT within this set. With an OT value as high as 5.7 ng/l_air,_ butyl acrylate revealed the highest OT value among all analysed n-alkyl acrylates. Since butyl acrylate and 1-octen-3-one showed similar odour qualities and a similar molecular structure, we expected their OT values to show similar trends within their respective homologous series. However, we unexpectedly observed an OT-peak in the case of butyl acrylate, whereas 1-octen-3-one revealed one of the lowest OT values within its respective homologous series.

The median OT values of the investigated 1-alken-3-ones (set 4) fell within the range from 0.0032 to 55 ng/l_air_ being therefore generally lower than the values of their corresponding acrylate-analogues. However, the overall chain length appeared to have a greater influence on the OT values than observed in the acrylates. The lowest OT value was recorded for the shortest homologue 1-hexen-3-one, revealing values at least ten times lower than that of the other 1-alken-3-ones. Whereas increasing the chain length by one carbon atom resulted in an elevated OT value of 0.05 ng/l_air_ for 1-hepten-3-one, further elongation did not increase the OT values in the case of 1-octen-3-one (0.03 ng/l_air_) and 1-nonen-3-one (0.03 ng/l_air_) further. However, 1-alken-3-ones with a chain length above C_9_ showed a noticeable increase of OT values with increasing chain length resulting in values of 0.72 ng/l_air_ for 1-decen-3-one, 1.9 ng/l_air_ for 1-undecen-3-one, and 55 ng/l_air_ for 1-dodecen-3-one. Previous studies reported OT values that were by one order of magnitude higher for 1-hexen-3-one (0.02–0.04 ng/l_air_ [18, 33]) and 1-decen-3-one (0.08 ng/l_air_ [18]), but on the other hand, lower OT values for 1-nonen-3-one were provided (0.008 ng/l_air_ [8, 18]). Albeit, we could show in several studies that odour thresholds may vary to a major extent between subjects [18, 34, 35]. These differences in the OT values, or rather their mean or median values derived therefrom, are most likely linked to inter-individual differences in the sensitivity in the respective test panels and could potentially be minimized using larger panel sizes. However, this would necessitate novel test protocols to be developed that align the main advantages of the laborious and time-consuming odour threshold determination by GC-O, thus eliminating potential artefacts from impurities and working matrix-free, and the possibility of high-throughput testing. In our previous study, the odour threshold values were determined by two panellists [18], whereas now, we expanded the panel to up to seven panellists. Accordingly, the individual performance of every panellist should be of higher impact in smaller cohorts with regard to the median or mean OT value meaning that results from different groups or even the same research group may vary to a considerable extent, the smaller the panellist numbers. To account for this, we carried out this laborious approach of odour threshold determination in this study with a larger subject group, to gain a more comprehensive data set for comparison between the 1-alken-3-ones and the analogous acrylates. Thereby, we could, nevertheless, show high congruency of the obtained data from our previous and the present study with respect to 1-octen-3-one (0.03–1.12 ng/l_air_ [18, 36, 37]), 1-undecen-3-one (0.15 ng/l_air_ [18]), and 1-dodecen-3-one (33 ng/l_air_ [18]). Whereas the median odour thresholds did not correspond with any other literature data for some of the analysed substances, a strong increase in OT values could be, nevertheless, confirmed for 1-alken-3-ones with a chain-length of C11 or higher in both our studies, the current and our previous investigation [18].

#### Introduction of terminal double bonds

Overall, the introduction of a second terminal double bond in acrylates (set 2) did not lead to a significant increase or decrease of median OT values. The strongest influence was observed in 4-pentenyl acrylate that showed the lowest odour threshold (0.44 ng/l_air_) within set 2. In comparison to its saturated analogue pentyl acrylate (1.9 ng/l_air_), the introduction of a terminal double bond led to a decrease of the OT by a factor of 4. However, the contrary effect could be observed in 3-butenyl acrylate (7.4 ng/l_air_), which was the least odour active substance in set 2. The second terminal double bond led to a slightly higher odour threshold value than in butyl acrylate (5.7 ng/l_air_).

The introduction of a second terminal double bond affected each substance of set 5 to a different degree and to a lager extent than in their corresponding acrylates. In the case of 1,8-nonadien-3-one (0.12 ng/l_air_) and 1,7-octadien-3-one (0.92 ng/l_air_), the desaturation resulted in an increase of their OT values by the factor 4 and 32, respectively. Since both of their monounsaturated analogues, 1-nonen-3-one and 1-octen-3-one, showed approximately the same OT values (0,030 ng/l_air_ and 0,029 ng/l_air_), it is surprising that the introduction of a second terminal double bond had a more pronounced effect on 1,7-octadien-3-one then on 1,8-nonadien-3-one. Since panellists showed a highly differing sensitivity towards 1,8-nonadien-3-one, the effect of the second double bond might appear to be weaker when interpreting the median OT values. However, opposite effects on the odour activity could be observed in 1,9-decadien-3-one. Although its median OT value of 0.23 ng/l_air_ lay in between the values of 1,8-nonadien-3-one and 1,7-octadien-3-one, its OT value was lower than the OT value of its monounsaturated analogue (1-decadien-3-one), revealing the opposite effect of what was observed for 1,7-octadien-3-one and 1,8-nonadien-3-one. Since 1,7-octadien-3-one, 1,8-nonadien-3-one, and 1,9-decadien-3-one showed median OT values between 0.12 and 0.92 ng/l_air_, and therefore values within a low deviation range by the factor of 7, it was concluded that the odour activity of set 5 was not affected by the change in chain length in the same way as it was observed for their monounsaturated analogues (set 1), but rather by the presence of a second terminal double bond.

Overall, the alkadien-3-ones of set 5 showed lower median OT values than their respective acrylate analogues, confirming that acrylates are less odour active than their respective ketones. Comparing the OT values of sample sets 2 and 5, it became apparent that in both sets, the C_8_ substance (3-butenyl acrylate and 1,7-octadien-3-one) showed the highest median OT value and the C_9_ substances (4-pentenyl acrylate and 1,8-nonadien-3-one) the lowest, while the C_10_ compounds (5-hexenyl acrylate and 1,9-decadien-3-one) yielded values in between.

#### Influence of shifting double bonds on the OT values

Within set 3, 5-hexenyl acrylate and (*E*)-2-hexenyl acrylate were the least odour active compounds showing OT values of 2.8 ng/l_air_ and 0.99 ng/l_air_, respectively. In comparison to their saturated analogue hexyl acrylate (2.9 ng/l_air_), both the introduction of a terminal double bond and a double bond in close proximity to the ester group resulted in no or only a minor decrease in the respective OT values. However, if the double bond was introduced between those positions, as it was the case for (*E*)-4-hexenyl acrylate (0.044 ng/l_air_) and (*E*)-3-hexenyl acrylate (0,014 ng/l_air_), the additional double bond yielded noticeably lower OT values, being decreased by a factor of 72 and 286, respectively, compared to hexyl acrylate. Thereby, (*E*)-3-hexenyl acrylate showed, with a median OT value of 0.014 ng/l_air_, the second lowest OT value among all investigated substances, and moreover, the lowest OT value among all analysed acrylates within this study. It is interesting to note that double bonds in position 3 and especially those in (*Z*)-configuration appear to be of special smell impact in straight-chain carbonyl compounds. Similar observations can be made for unsaturated aldehydes, alcohols, and ketones [18, 34, 38]. This gives rise to the assumption that this structural feature might exert a specific interaction with receptor structures. One might consider in this respect the potential formation of complex structures that may arise, for example, with a Zn ion-moiety that has been reported to be an integral part of odorant receptor structures. This might further explain why double bonds in closer proximity (in position 2) or farther distance (terminal end of the chain) do not exert this effect.

Within set 6, represented by bis-unsaturated decadien-3-ones (no. 23–26), the lowest median OT values were detected for 1,9-decadien-3-one (0.23 ng/l_air_), which contained two terminal double bonds, as well as (*E*)-1,6-decadien-3-one (0.37 ng/l_air_), whose second double bond was located closest to the carbonyl function. In both cases, the introduction of a second double bond only led to a minor decrease of median OT values, when compared to 1-decan-3-one (0.72 ng/l_air_). As the same effect was observed in the corresponding acrylates, the introduction of these double bonds appeared to have no major effect on the odour activity of these molecules. However, if a second double bond between these positions was introduced, as it was the case for (*E*)-1,7-decadien-3-one (5.6 ng/l_air_) and (*E*)-1,8-decadien-3-one (7.8 ng/l_air_), elevated OT values were obtained, gaining thus the opposite effect than in the corresponding acrylates. These results suggest that additional ester bond in the acrylates might have an influence on the odour activity either by synergistic effects caused by the ester group or the oxygen located inside the carbon chain or even by enzyme-induced cleavage of the ester bond in the nasal epithelium (with reference to potential perireceptor events).

#### Individual variances in perception

The individual OT values of the panellists showed low to medium variation for 13 of the 26 analysed substances. In those cases, the individual odour threshold values differed by a factor of 4–20 between the highest and the lowest value. Overall, the range of variation was smaller in case of the acrylates, with factors that were generally below 16. Thereby, the highest range of variation with factors of 510 and 500 between minimum and maximum value was observed for 1-undecen-3-one (no. 19; 0.48–245 ng/l_air_) and 1,8-nonadien-3-one (no. 22; 0.015–7.5 ng/l_air_). Interestingly, this was caused by a single panellist who was obviously extraordinarily sensitive to these substances, and one or two panellist, in case of 1-undecen-3-one and 1,8-nonadien-3-one, who were very insensitive. The individual OT values of the remaining panellists ranged within a factor of 7 for 1-undecen-3-one and 4 for 1,8-nonadien-3-one. The corresponding acrylates did not show such pronounced differences. Accordingly, the pattern of receptor activation is likely to deviate to some extent for the acrylates and ketones.

The individual differences in smell perception in humans can be caused by several factors. First, volatile compounds that enter the nasal cavity can undergo biotransformation processes in the course of the so-called perireceptor events, meaning that they can undergo metabolization. One of the potentially involved systems there is the cytochrome P450 family that is highly expressed in the nasal mucus layer. The expression profiles of these enzymes are different in every individual and might therefore lead to differences in the perception of smell [39–42]. Since the tested acrylates contain an ester group as well as a double bond moiety, and accordingly groups that can be attacked by enzymes, these molecules are presumably more prone to to such transformations. This can serve as possible explanation for deviating processes in the corresponding ketones as only the keto function may be modified and there most likely in the frame of a redox reaction. Second, it has been shown that receptor expression can likewise deviate between subjects, and moreover substances may activate more than one receptor, or one receptor may be activated by different substances, and their derivatives [43, 44]. The resulting potential for sensory deviation is, accordingly, vast and can impact smell character and intensity in a given subject to a major extent. In view of this, it is even surprising that there were yet such striking correlations in smell perception between some of the investigated acrylates and corresponding enones. This would raise the question if these substances are of special meaning to us. Even if this might appear to be a rather philosophical question—it surely is not when considering the quasi-ubiquitous appearance of some of these compounds. Oct-1-en-3-one and (*Z*)-1,5-octadien-3-one are considered as degradation products of common unsaturated acids, as smell constituents of any type of food or natural smell, and as potent constituents of the smell of meat and blood; surely there is some meaning to these. Understandably, this would justify enough meaning to translate this potent smell percept into modern world smells via cross-activation that nature never intended to develop via evolutionary processes. It will be interesting to further explore modern smells originating from a synthetic world in this respect.

## Conclusion

In the present study, 13 acrylic esters and 13 1-alken-3-ones/alkadien-3-ones were analysed with regard to their chromatographic behaviour, their odour qualities, and their odour thresholds. To this aim, 18 substances were specifically synthesized and analysed by a combinatory sensory-analytical approach that eliminates matrix effects and rules out the potential of artefacts due to odorous side-products.

Overall, the investigated substances showed *mushroom-like*, *geranium-like*, or *fruity* odour qualities. However, short chained molecules, namely ethyl acrylate, propyl acrylate, and 1-hexen-3-one, elicited *geranium-like*, *lighter gas-like*, *garlic-like*, and *glue-like* odour qualities. Accordingly, we could show that the odour quality depended on the overall chain length in both substance classes, even though this effect was more pronounced in the series of the 1-alken-3-ones. It was found that butyl acrylate and 1-octen-3-one, as well as hexyl acrylate and 1-decadien-3-one, revealed similar odour qualities, whereas the other pairs rather deviated in their smell properties. Accordingly, only partial congruency in the activation patterns of the respective receptor systems would be likely. The results suggest that the introduction of additional double bonds did not have a pronounced effect on the odour qualities of alkadien-3-ones and their analogue acrylates. However, if a second double bond was introduced closer to the carbonyl moiety but not yet too close as it was the case for (*E*)-1,8-decadien-3-one and (*E*)-1,7-decadien-3-one, the odour qualities changed noticeably.

The results show that most investigated substances exhibit low odour thresholds and therefore high odour potencies. Overall, the investigated acrylates appear to be less odour active than their corresponding enones. In this study, the lowest OT value was detected for 1-hexen-3-one and the highest OT value for 1-dodecen-3-one. While 1-alken-3-ones showed a higher dependency on the chain length itself, their corresponding acrylates provided more constant OT values with increasing chain length. It became thus apparent that the acrylic function itself influenced the odour threshold to a lager extent than the carbonyl moiety. The introduction of a second terminal double bond did neither lead to a clear increase nor a clear decrease of the odour thresholds both in the acrylates (set 2) and the alkadien-3-ones (set 5). However, the results suggest that the OT of terminal bis-unsaturated alkadien-3-ones is linked rather to the presence of a second terminal double bond than the chain length. Surprisingly, the introduction of a second double bond that was closer to the alkyl or acrylate moiety but not yet too close led to a noticeable effect in both sets. Whereas the OT values in the cases of (*E*)-3-hexenyl acrylate and (*E*)-4-hexenyl acrylate were decreased when compared to their respective isomers, the OT values of (*E*)-1,8-decadien-3-one and (*E*)-1,7-decadien-3-one were elevated. In both substance classes, these odorants showed differing OT values from the remaining compounds comprising their homologous series, although the effect was different for the acrylates (increase in odour activity) as it was for the enones (decrease in odour activity). Our study reveals that despite similarities in smell there are yet clear and pronounced sensory effects induced by specific molecular moieties. These may be even more pronounced when changing to different atomic constituents such as sulphur. Further investigation covering a greater range of similar substance classes, e.g. thioester structures, will help to generate an even deeper understanding of such effects.

Summarizing the results of the present study, the provided chromatographic and analytical data, together with the sensory information comprising odour qualities and the respective odour thresholds in air, will add to our knowledge of how we perceive smell in our modern world and will support future elucidation of smell effects both in food and non-food materials.

## Supplementary Information

Below is the link to the electronic supplementary material.

Supplementary file1 (DOCX 494 kb)

## References

[1] Strategy WM. The Global Acrylates Marke2019. Report No.: ID: 4665234.

[2] Bauer P, Buettner A. Characterization of odorous and potentially harmful substances in artists' acrylic paint. front public health. 2018;6:350. 10.3389/fpubh.2018.00350. 10.3389/fpubh.2018.00350 PMC6281683 30555813

[3] Denk P, Buettner A (2017). Sensory characterization and identification of odorous constituents in acrylic adhesives. Int J Adhes Adhes.

[4] Bauer P, Denk P, Fuss JM, Lorber K, Ortner E, Buettner A (2019). Correlations between odour activity and the structural modifications of acrylates. Anal Bioanal Chem.

[5] Lopez Pinar A, Rauhut D, Ruehl E, Buettner A (2016). Effects of Botrytis cinerea and Erysiphe necator fungi on the aroma character of grape must: a comparative approach. Food Chem.

[6] Niebler J, Farran D, Roussel C, Balaban TS, Buettner A, editors. Synthesis of new thioterpenoid odorants with unexpected mushroom-like flavour qualities. Current topics in flavor chemistry & biology. Proceedings of the 10th Wartburg Symposium on Flavor Chemistry & Biology 2014; Eisenach, Germany.

[7] Widder S, Sen A, Grosch W (1991). Changes in the flavour of butter oil during storage. Zeitschrift für Lebensmittel-Untersuchung und Forschung.

[8] Reiners J, Grosch W (1998). Odorants of virgin olive oils with different flavor profiles. J Agric Food Chem.

[9] Blank I, Grosch W. Evaluation of potent odorants in dill seed and dill herb (Anethum graveolens L.) by aroma extract dilution analysis. J Food Sci. 1991;56(1):63–7. 10.1111/j.1365-2621.1991.tb07976.x.

[10] Georgilopoulos DN, Gallois AN (1987). Volatile flavour compounds in heated blackberry juices. Zeitschrift für Lebensmittel-Untersuchung und Forschung.

[11] Buttery RG, Guadagni DG, Ling LC (1978). Volatile aroma components of cooked artichoke. J Agric Food Chem.

[12] Frauendorfer F, Schieberle P (2019). Key aroma compounds in fermented Forastero cocoa beans and changes induced by roasting. Eur Food Res Technol.

[13] Aisala H, Sola J, Hopia A, Linderborg KM, Sandell M (2019). Odor-contributing volatile compounds of wild edible Nordic mushrooms analyzed with HS-SPME-GC-MS and HS-SPME-GC-O/FID. Food Chem.

[14] Ott A, Fay LB, Chaintreau A (1997). Determination and origin of the aroma impact compounds of yogurt flavor. J Agric Food Chem.

[15] Roberts DD, Acree TE (1996). Effects of heating and cream addition on fresh raspberry aroma using a retronasal aroma simulator and gas chromatography olfactometry. J Agric Food Chem.

[16] Pons M, Dauphin B, La Guerche S, Pons A, Lavigne-Cruege V, Shinkaruk S (2011). Identification of impact odorants contributing to fresh mushroom off-flavor in wines: incidence of their reactivity with nitrogen compounds on the decrease of the olfactory defect. J Agric Food Chem.

[17] Dunkel A, Steinhaus M, Kotthoff M, Nowak B, Krautwurst D, Schieberle P (2014). Genuine Geruchssignaturen der Natur – Perspektiven aus der Lebensmittelchemie für die Biotechnologie. Angew Chem.

[18] Lorber K, Schieberle P, Buettner A (2014). Influence of the chemical structure on odor qualities and odor thresholds in homologous series of alka-1,5-dien-3-ones, alk-1-en-3-ones, alka-1,5-dien-3-ols, and alk-1-en-3-ols. J Agric Food Chem.

[19] Kocak A, Bagci E, Bakoglu A. Chemical composition of essential oils of Achillea teretifolia Willd. and A. millefolium L. subsp. millefolium growing in Turkey. Asian Journal of Chemistry. 2010;22(5):3653–8.

[20] Denzer MY, Gailer S, Kern DW, Schumm LP, Thuerauf N, Kornhuber J (2014). Quantitative Validation of the n-Butanol Sniffin' Sticks Threshold Pens. Chemosens Percept.

[21] Beauchamp J, Frasnelli J, Buettner A, Scheibe M, Hansel A, Hummel T. Characterization of an olfactometer by proton-transfer-reaction mass spectrometry. Meas Sci Technol. 2010;21(2). 10.1088/0957-0233/21/2/025801.

[22] Beauchamp J, Scheibe M, Hummel T, Buettner A (2014). Intranasal odorant concentrations in relation to sniff behavior. Chem Biodivers.

[23] van Den Dool H, Dec. Kratz P. A generalization of the retention index system including linear temperature programmed gas—liquid partition chromatography. Journal of Chromatography A. 1963;11:463–71. doi:10.1016/s0021-9673(01)80947-x. 10.1016/s0021-9673(01)80947-x 14062605

[24] Ullrich F, Grosch W (1987). Identification of the most intense volatile flavour compounds formed during autoxidation of linoleic acid. Zeitschrift für Lebensmittel-Untersuchung und Forschung.

[25] Xiao Q, He Q, Li J, Wang J. 1,4-Diazabicyclo[2.2.2]octane-promoted aminotrifluoromethylthiolation of alpha,beta-unsaturated carbonyl compounds: N-Trifluoromethylthio-4-nitrophthalimide acts as both the nitrogen and SCF3 sources. Org Lett. 2015;17(24):6090–3. 10.1021/acs.orglett.5b03116. 10.1021/acs.orglett.5b03116 26655102

[26] van den Nieuwendijk Adrianus MCH, Kriek Nicole MAJ, Brussee J, van Boom JH, van der Gen A (2000). Stereoselective synthesis of (2R,5R)- and (2S,5R)-5-hydroxylysine. Eur J Org Chem.

[27] Vakhidov RR, Alekseev SB (2011). Synthesis of 5Z,9E-tridecadien-1-ylacetate, an attractant of Trichoplusia ni. Chem Nat Compd.

[28] Brown DC, Nichols SA, Gilpin AB, Thompson DW. Transition metal promoted alkylations of unsaturated alcohols. Alkylation of alkynols with organoalanes promoted by Group IVA metal-cyclopentadienyl compounds. J Org Chem. 1979;44(20):3457–61. 10.1021/jo01334a002.

[29] Hara S, Kishimura K, Suzuki A. Reaction of organoboranes with the dianion of phenoxyacetic acid. The first direct synthesis of carboxylic acids from organoboranes. Tetrahedron Letters. 1978;19(32):2891–994. 10.1016/s0040-4039(01)94891-9.

[30] Buettner A, Beauchamp J (2010). Chemical input – sensory output: diverse modes of physiology–flavour interaction. Food Qual Prefer.

[31] Beauchamp J, Frasnelli J, Buettner A, Scheibe M, Hansel A, Hummel T. PTR-MS Characterisation of an olfactometer. 4th International Conference on Proton Transfer Reaction Mass Spectrometry and its Applications 2009.; Obergurgl, Austria: Innsbruck University Press; 2009. p. 186–90.

[32] Schilling B, Buettner A (2017). Springer Handbook of Odor. Springer Handbooks.

[33] Blank I, Fischer K-H, Grosch W. Intensive neutral odorants of linden honey. Differences from honeys of other botanical origin. 1989.

[34] Lorber K, Buettner A (2015). Structure-odor relationships of (E)-3-alkenoic acids, (E)-3-alken-1-ols, and (E)-3-alkenals. J Agric Food Chem.

[35] Elsharif SA, Buettner A (2018). Structure-odor relationship study on geraniol, nerol, and their synthesized oxygenated derivatives. J Agric Food Chem.

[36] Boerger D, Buettner A, Schieberle P. State-of-the-Art in Flavour Chemistry and Biology. Eisenach, Germany: Deutsche Forschungsanstalt für Lebensmittelchemie; 2005.

[37] Guth H, Grosch W (1990). Comparison of stored soya-bean and rapeseed oils by aroma extract dilution analysis. Lebensmittel-Wissenschaft & Technologie.

[38] Lorber K, Zeh G, Regler J, Buettner A (2018). Structure-odor relationships of ( Z)-3-alken-1-ols, ( Z)-3-alkenals, and ( z)-3-alkenoic acids. J Agric Food Chem.

[39] Zhang X, Zhang QY, Liu D, Su T, Weng Y, Ling G (2005). Expression of cytochrome p450 and other biotransformation genes in fetal and adult human nasal mucosa. Drug Metab Dispos.

[40] Chougnet A, Woggon WD, Locher E, Schilling B (2009). Synthesis and in vitro activity of heterocyclic inhibitors of CYP2A6 and CYP2A13, two cytochrome P450 enzymes present in the respiratory tract. ChemBioChem.

[41] Nagashima A, Touhara K (2010). Enzymatic conversion of odorants in nasal mucus affects olfactory glomerular activation patterns and odor perception. J Neurosci.

[42] Schilling B, Kaiser R, Natsch A, Gautschi M (2009). Investigation of odors in the fragrance industry. Chemoecology.

[43] Araneda RC, Kini AD, Firestein S (2000). The molecular receptive range of an odorant receptor. Nat Neurosci.

[44] Katada S, Hirokawa T, Oka Y, Suwa M, Touhara K (2005). Structural basis for a broad but selective ligand spectrum of a mouse olfactory receptor: mapping the odorant-binding site. J Neurosci.

[45] Davy K, Braden M (1991). Residual monomer in acrylic polymers. Biomaterials.

[46] Borges RM, Ranganathan Y, Krishnan A, Ghara M, Pramanik G (2011). When should fig fruit produce volatiles? Pattern in a ripening process. Acta Oecologica.

[47] Yang YQ, Yin HX, Yuan HB, Jiang YW, Dong CW, Deng YL (2018). Characterization of the volatile components in green tea by IRAE-HS-SPME/GC-MS combined with multivariate analysis. PLoS ONE.

[48] Xu L, Fang X, Wu W, Chen H, Mu H, Gao H (2019). Effects of high-temperature pre-drying on the quality of air-dried shiitake mushrooms (Lentinula edodes). Food Chem.

[49] Tang K, Xi YR, Ma Y, Zhang HN, Xu Y. Chemical and sensory characterization of cabernet sauvignon wines from the Chinese Loess Plateau region. Molecules. 2019;24(6). doi:10.3390/molecules24061122. 10.3390/molecules24061122 PMC6471551 30901866

[50] Miyazato H (2018). Volatile composition and the key aroma compounds of the Citrus tachibana (Makino) Tanaka peel essential oil. Journal of Essential Oil Bearing Plants.

[51] Obretenov T, Hadjieva P (1977). Gas-chromatographic-mass-spectral analysis of aroma-compounds of bread. Z Lebensm Unters Forsch.

