# Supplementary material for: Influence of elongation and desaturation on chemosensory properties in acrylates and their corresponding 1-alken-3-ones
Source: Anal Bioanal Chem. 2022 Sep 22;414(28):8009–22. doi: 10.1007/s00216-022-04332-9 (PMC9613748; doi:10.1007/s00216-022-04332-9)
Supplement: Supplementary file 1 — Supplementary file1 (DOCX 494 kb) [file 216_2022_4332_MOESM1_ESM.docx]

**Analytical and Bioanalytical Chemistry**

**Influence of elongation and desaturation on chemosensory properties in acrylates and their corresponding 1-alken-3-ones**

Patrick Bauer^1,2^, Eva Ortner^2^, Andrea Buettner^1,2^

^1^ Friedrich-Alexander-Universität Erlangen-Nürnberg (FAU), Department of Chemistry and Pharmacy, Chair for Aroma and Smell Research, Henkestraße 9, 91054 Erlangen, Germany

^2^ Fraunhofer Institute for Process Engineering and Packaging (IVV), Giggenhauser Straße 35, 85354 Freising, Germany

*Address for correspondence
Phone +49 8161 491-715

E-mail [andrea.buettner@ivv.fraunhofer.de](mailto:andrea.buettner@ivv.fraunhofer.de)

1. Material and Methods

Syntheses

Within this study, 18 substances were synthesised according to literature procedures and characterised in detail regarding their chemosensory properties for the first time. The purity and identity of all synthesised products was ensured by ^1^H-NMR and GC-MS. Synthesis pathways are summarized in figures 1 and 2.

1.1 Synthesis of acrylates

All acrylates were synthesised according to the following procedure: Triethylamine (9.0 mmol, 0.91 g) and the respective alcohol (6.0 mmol) were dissolved in dichloromethane (DCM, 10 ml), and cooled in an ice-water bath to 0 °C. Acryloyl chloride (7.2 mmol, 0.65 g) was added dropwise, and the mixture was stirred for five minutes before the ice bath was removed. The mixture was then allowed to warm to room temperature and was stirred overnight. The reaction was terminated via the addition of 2 ml of water. The phases were separated, and the organic phase was dried with anhydrous sodium sulfate. The organic solvent was removed under reduced pressure and the crude product was purified by column chromatography (silica gel; petroleum ether / ethyl acetate = 30:1) [1].

1.2. Synthesis of 1-hepten-3-one and 1-decen-3-one

A solution of the respective alcohol (10.0 mmol) in DCM (30 ml) was added dropwise to a suspension of Dess-Martin periodinane (DMP, 11.0 mmol, 4.67 g) in DCM (30 ml) under nitrogen atmosphere and was stirred at room temperature for 6 h. The reaction mixture was filtrated and washed with a solution of 0.5 M sodium thiosulfate pentahydrate in saturated sodium bicarbonate (40 ml), deionized water (30 ml) and brine (30 ml). The obtained organic phase was then dried over sodium sulfate. The solvent was removed under reduced pressure and purified by column chromatography (silica gel; petroleum ether / ethyl acetate = 30:1) [2].

1.3 Synthesis of 1,7-octen-3-one, 1,8-nonen-3-one, (*E*)-1,6-decadienone, (*E*)-1,7-decadienone, (*E*)-1,8-decadienone, (*E*)-1,9-decadienone and 1-dodecen-3-one

The majority of the bis-unsaturated ketones were synthesised starting from the corresponding alcohol that was lacking the vinyl moiety attached to the carbonyl function (see Figure 1 pathway e). The synthesis was started by adding a solution of the corresponding alcohol (15.0 mmol), dissolved in DCM (15 ml), dropwise to a suspension of DMP (15.0 mmol) in DCM (45 ml) under nitrogen atmosphere. After stirring the mixture at room temperature for 6 h, the reaction mixture was filtrated and consecutively washed with a solution of 0.5 M sodium thiosulfate pentahydrate in saturated sodium bicarbonate (60 ml), deionized water (45 ml) and brine (45 ml). The organic phase was then dried over sodium sulfate, and the solvent was removed under reduced pressure [2].

The obtained aldehydes (7 mmol) were then dissolved in anhydrous tetrahydrofuran (THF, 15 ml), and added dropwise into a vinylmagnesium bromide solution (1.0 M in THF, 7.5 ml) under nitrogen atmosphere at 0 °C. After stirring for 1 h, the mixture was warmed to r.t. and stirred overnight. The mixture was washed with 8 ml 10% hydrogen chloride solution, and the organic phase was extracted with diethyl ether (3 x 8 ml). The organic phase was then dried over sodium sulfate, and the solvent was removed under reduced pressure. The obtained alcohols were then further oxidised following the steps described above (2.8.2). However, we used 4.0 mmol of the corresponding alcohols and adapted the amounts of the other reactants accordingly.

1.4 Synthesis of the corresponding alcohol-reactants

Unlike other reactants, the alcohols needed for the preparation of (*E*)-1,6-decadienone, (*E*)-1,7-decadienone, (*E*)-1,8-decadienone were not commercially available and were therefore synthesised.

1.4.1 Synthesis of (*E*)-4-octenol

Triethylorthoacetate (20.24 g, 115.6 mmol) and 1-hexen-3-ol (3.37 g, 33.7 mmol) were dissolved in propionic acid (0.1 g, 0.83 mmol) and stirred at 135 °C for 2 h. After cooling to room temperature, the mixture was extracted with diethyl ether (30 ml), washed with saturated sodium hydrogen carbonate and sodium chloride solutions and dried over magnesium sulfate. After the solvent was removed under reduced pressure, the residue was purified via column chromatography (silica gel, hexane : ethyl acetate = 10:1) to obtain (*E*)-4-octenoic acid ethyl ester.

To obtain the desired product (*E*)-4-octenol, (*E*)-4-octenoic acid ethyl ester (4.35 g, 14.82 mmol) was added dropwise over 1 h to a cooled (0 °C) suspension of lithium aluminium hydride (0.98 g, 26.9 mmol) in anhydrous diethyl ether (30 ml) under nitrogen atmosphere. The mixture was then warmed to room temperature and stirred for 2 h. The solution was again cooled to 0 °C, and deionised water (15 ml) was added dropwise over 0.5 h. The solution was decanted and the aqueous phase was washed with diethyl ether (15 ml). The combined organic layers were washed with a saturated sodium chloride solution (2 x 15 ml) and dried over magnesium sulfate. The solvent was removed under reduced pressure and the residue was vacuum distilled to obtain (*E*)-4-octenol [3].

1.4.2 Synthesis of (*E*)-5-octenol

A solution of 5-hexyn-1-ol (4.91 g, 50.0 mmol) in DCM (20 ml) was added dropwise into a cooled (0 °C) solution of diethylaluminium chloride (55.1 mmol, 6.64 g) in DCM (35 ml) under nitrogen atmosphere and stirred for 3 h. Then, a solution of bis(cyclopentadienyl)titanium dichloride (2.54 g, 10.2 mmol) in DCM (20 ml) was added dropwise to the reaction mixture while maintaining temperature as well as the nitrogen atmosphere. The reaction was terminated by successively adding 8 ml of methanol and 20 ml of 5% sulfuric acid solution saturated with sodium chloride. The obtained solution was then stirred under oxygen atmosphere for 2 h and filtered over a bed of Celite. The solution was extracted with diethyl ether (2 x 30 ml) and the organic phase was dried over sodium sulfate and filtered [4]. After the solvent was removed under reduced pressure, the residue was purified via column chromatography (silica gel) using petroleum ether and ethyl acetate (10:1) as eluent.

1.4.3 Synthesis of (*E*)-6-octenol

A solution of 9-borabicyclo(3.3.1.)nonan (3.15 g, 25.0 mmol) in dry THF (50 ml) was added dropwise to cooled (0 °C) solution of 1,4-hexadiene (2.0 g, 24.34 mmol) in dry THF (25 ml) under nitrogen atmosphere and stirred at room temperature for 7 h (mixture I). Furthermore, reaction mixture II was prepared by adding a solution of phenoxyacetic acid (7.4 g, 49.0 mmol) in THF (40 ml) to a 2.0 M solution of lithium diisopropylamide in THF (50 ml) under nitrogen atmosphere at 0 °C. The mixture was stirred for 4 h at room temperature. Both mixtures were combined under nitrogen atmosphere at 0 °C and stirred at 66 °C overnight. Then, the mixture was cooled again to 0 °C and 3 M NaOH (25 ml), followed by a 45% H_2_O_2_ solution (22 ml) were successively added slowly. After stirring for 3 h, the mixture was washed with diethyl ether. The aqueous layer was acidified with a 5% HCl solution (10 ml) and then washed with diethyl ether. The combined organic phases were washed with brine (15 ml) and dried over sodium sulfate. After removing the solvent, the residue was purified on silica gel (petroleum ether : ethyl acetate = 10:1) to obtain (*E*)-6-octenoic acid as a brown oil [5].

To obtain the desired reactant (*E*)-6-octen-1-ol, a solution of lithium aluminium hydride (0.51 g, 13.42 mmol) in diethyl ether (15 ml) was prepared and cooled to 0 °C. Then, a solution of (*E*)-6-octenoic (1.73 g, 12.2 mmol) in 10 ml diethyl ether was added dropwise and the mixture was stirred at room temperature for 1 h. The solution was then washed successively with deionized water (10 ml), a 10% H_2_SO_4_ solution (8 ml) and was then extracted with diethyl ether (3 x 10 ml). The mixture was dried over sodium sulfate, and the solvent was removed under reduced pressure to obtain (*E*)-6-octen-1-ol.

**2, NMR spectroscopy**

**Pentyl acrylate**: M = 142.2 g/mol. Yield = 515.42 mg (3.62 mmol, 60.41%), purity 95.7% (GC)

**MS-EI,** *m/z* (relative intensity in %): 55 (99.9), 70 (58.4), 73 (45.2), 42 (16.4), 41 (10.3), 43 (8.4), 69 (7.6), 85 (7.0), 56 (5.4), 71 (3.6)

**^1^H-NMR** (600 MHz, CDCl_3,_ room temperature): δ [ppm] = 6.40 (dd, J = 2.0 Hz, J’ = 17.2 Hz, 1H, **1**), 6.12 (dd, J = 12.2 Hz, J‘ = 17.1 Hz, 1H, **3**), 5.82 (dd, J = 2.1 Hz, J‘ = 16.8 Hz, 1H, **2**), 3.97 (t, J = 7.0 Hz, 2H, **4**), 1.60 (m, J = 7.1Hz, 2H, **5**), 1.40 (m, 4H, **6** and **7**), 0.9 (t, J = 8.0 Hz, 3H, **8**) ppm


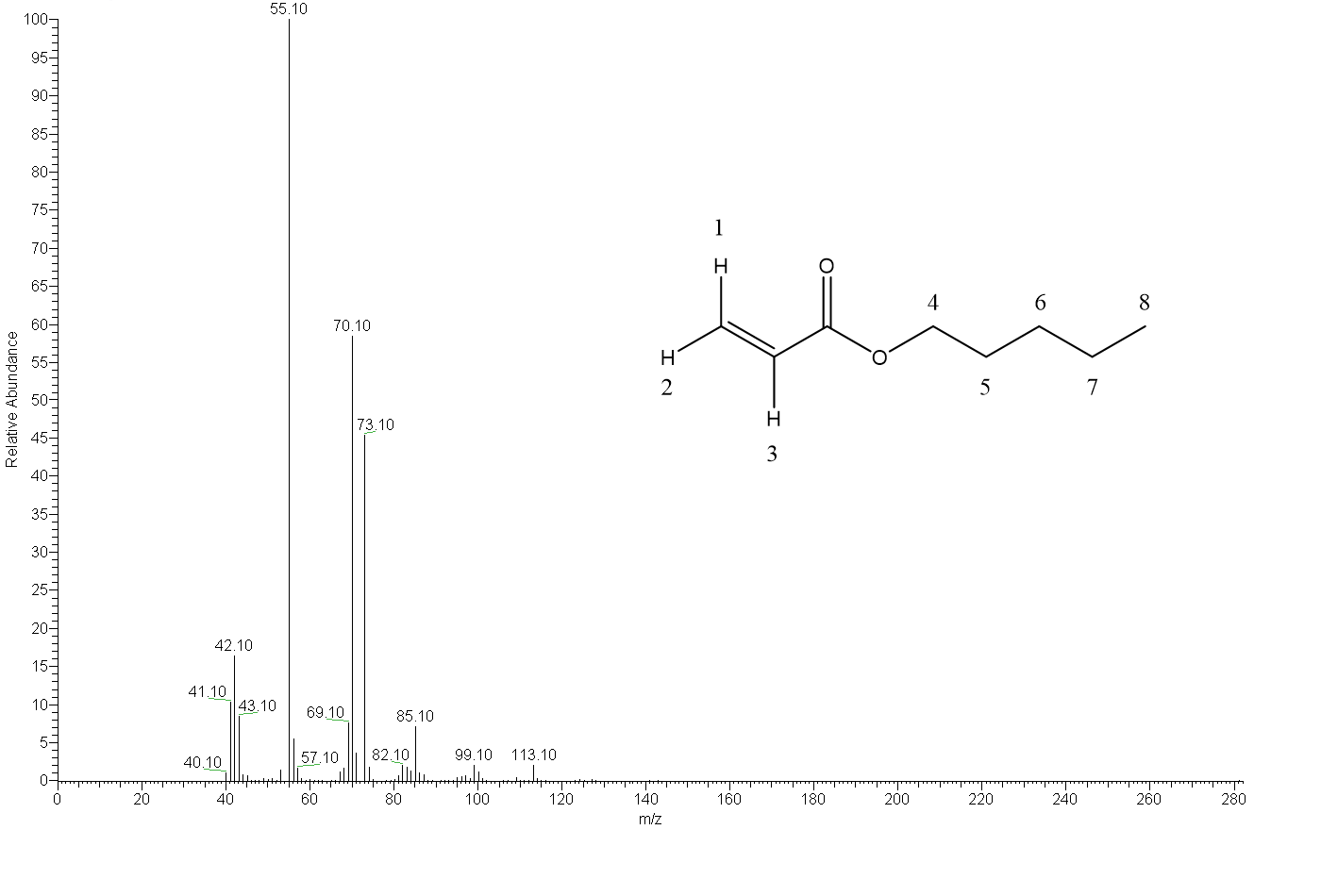


**Heptyl acrylate** M = 170.25 g/mol. Yield = 645.69 mg (3.79 mmol, 63.21%), purity 98.7% (GC)

**MS-EI,** *m/z* (relative intensity in %): 55 (99.9), 70 (48.3), 73 (45.5), 56 (43.8), 98 (27.4), 69 (25.9), 41 (20.5), 57 (14.0), 42 (10.6), 43 (10.3)

**^1^H-NMR** (600 MHz, CDCl_3,_ room temperature): δ [ppm] = 6.41 (dd, J = 2.1 Hz, J’ = 17.2 Hz, 1H, **1**), 6.12 (dd, J = 12.3 Hz, J‘ = 17.1 Hz, 1H; **3**), δ = 5.83 (dd, J = 2.0 Hz, J’ = 16.8 Hz, 1H, **2**), 3.97 (t, J = 7.1Hz, 2H, **4**), 1.60 (p, J = 7.2 Hz, 2H, **5**), 1.43 (m, J = 7.3 Hz, 2H, **6**), 1.26-12.4 (m, 6H, **7-9**), 0.88 (t, J = 8.0 Hz, 3H, **10**) ppm


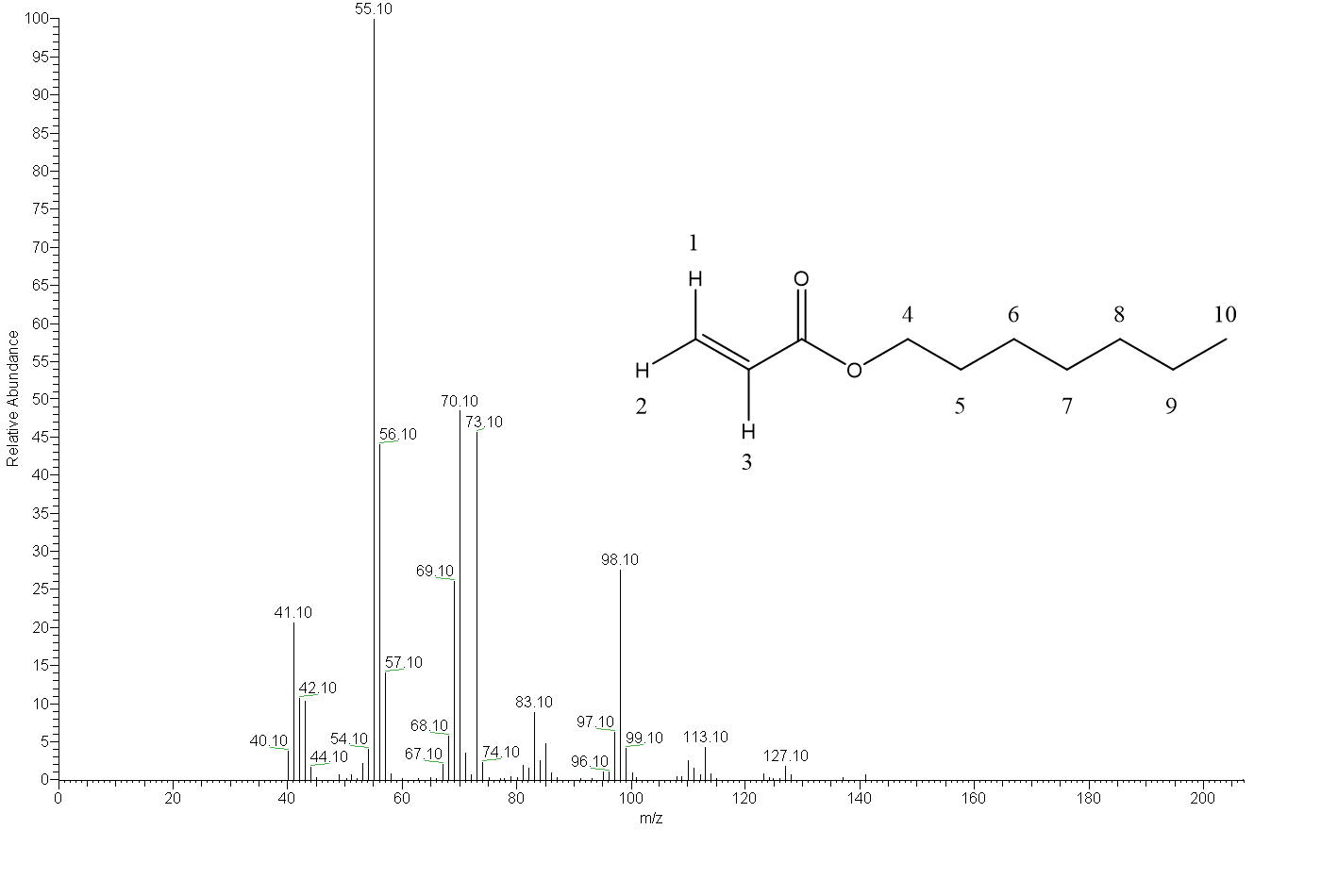


**Octyl acrylate** M = 184.28 g/mol. Yield = 792.33 mg (4.30 mmol, 71.66%), purity 98.5% (GC)

**MS-EI,** *m/z* (relative intensity in %): 55 (99.9), 73 (46.7), 70 (44.2), 56 (37.2), 83 (36.4), 84 (34.0), 69 (33.0), 41 (20.9), 43 (18.4), 112 (17.6)

**^1^H-NMR** (600 MHz, CDCl_3,_ room temperature): δ [ppm] = 6.41 (dd, J = 2.1 Hz, J’ = 17.2 Hz, 1H, **1**), 6.12 (dd, J = 12.3 Hz, J‘ = 17.1 Hz, 1H, **3**), δ = 5.83 (dd, J = 2.0 Hz, J’ = 16.8 Hz, 1H, **2**), 3.97 (t, J = 7.1Hz, 2H, **4**), 1.60 (p, J = 7.2 Hz, 2H, **5**), 1.43 (p, J = 7.3 Hz, 2H, **6**), 1.32 -12.6 (m, 8H, **7-10**), 0.88 (t, J = 8.0 Hz, 3H, **11**) ppm


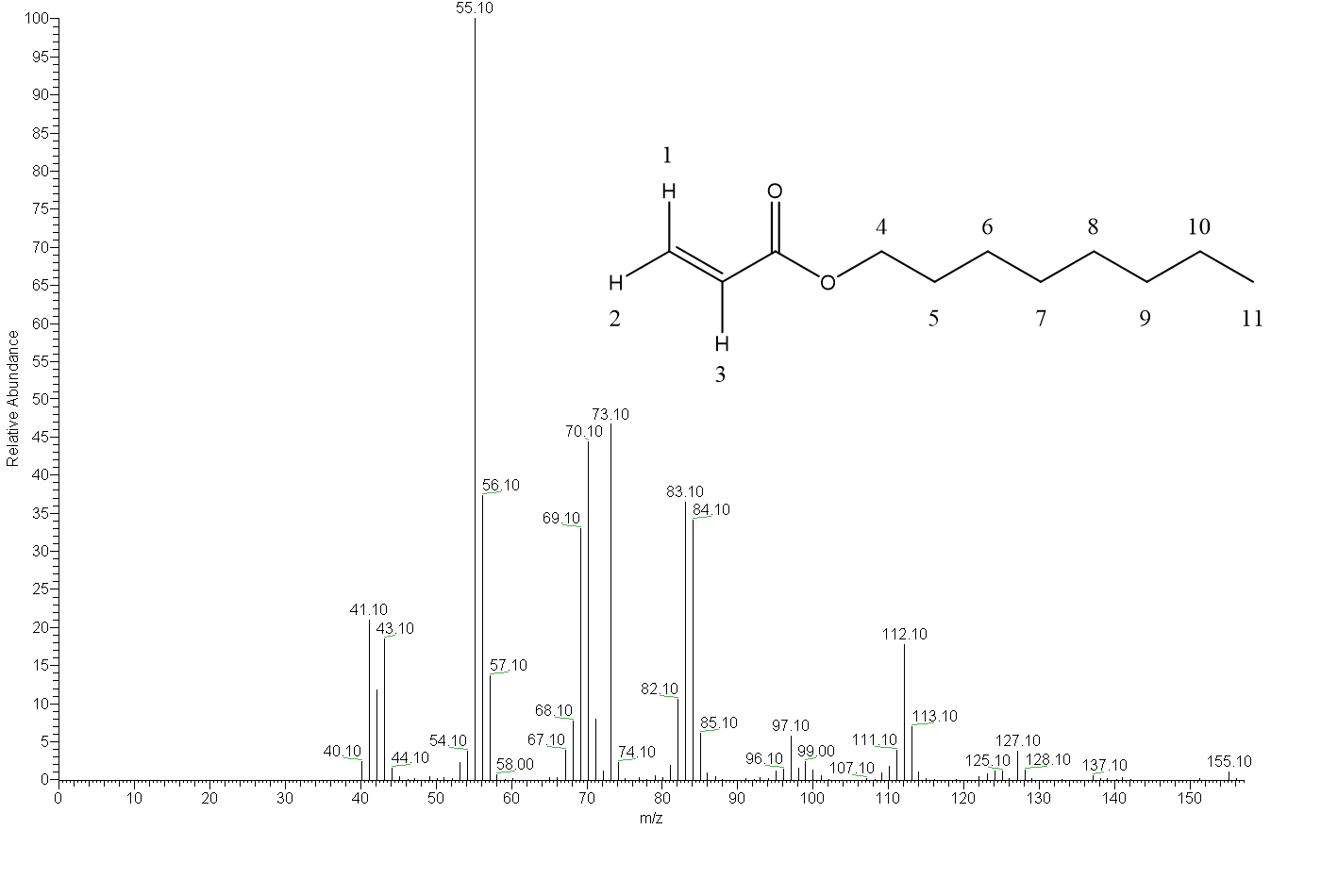


**3-Butenyl acrylate** M = 126.15 g/mol. Yield = 433.93 mg (3.44 mmol, 57.33%), purity 97.8 % (GC)

**MS-EI,** *m/z* (relative intensity in %): 55 (99.9), 54 (46.3), 85 (13.7), 56 (3.3), 68 (3.2), 53 (3.1), 41 (2.1), 40 (1.6), 84 (1.6), 86 (1.5)

**^1^H-NMR** (600 MHz, CDCl_3,_ room temperature): δ [ppm] = 6.41 (dd, J = 2.1 Hz, J’ = 17.2 Hz, 1H, **1**), 6.12 (dd, J = 12.2 Hz, J‘ = 16.8 Hz, 1H, **3**), 5.83 - 5.82 (m, 2H, **2** and **6**), 5.13 (d, J = 9.1 Hz, 1H, **8**), 4.88 (d, J = 8.3 Hz, 1H, **7**), 4.19 (t, J = 7.0 Hz, 2H, **4**), 2.24 (q, J = 7.1Hz,1H, **5**) ppm.


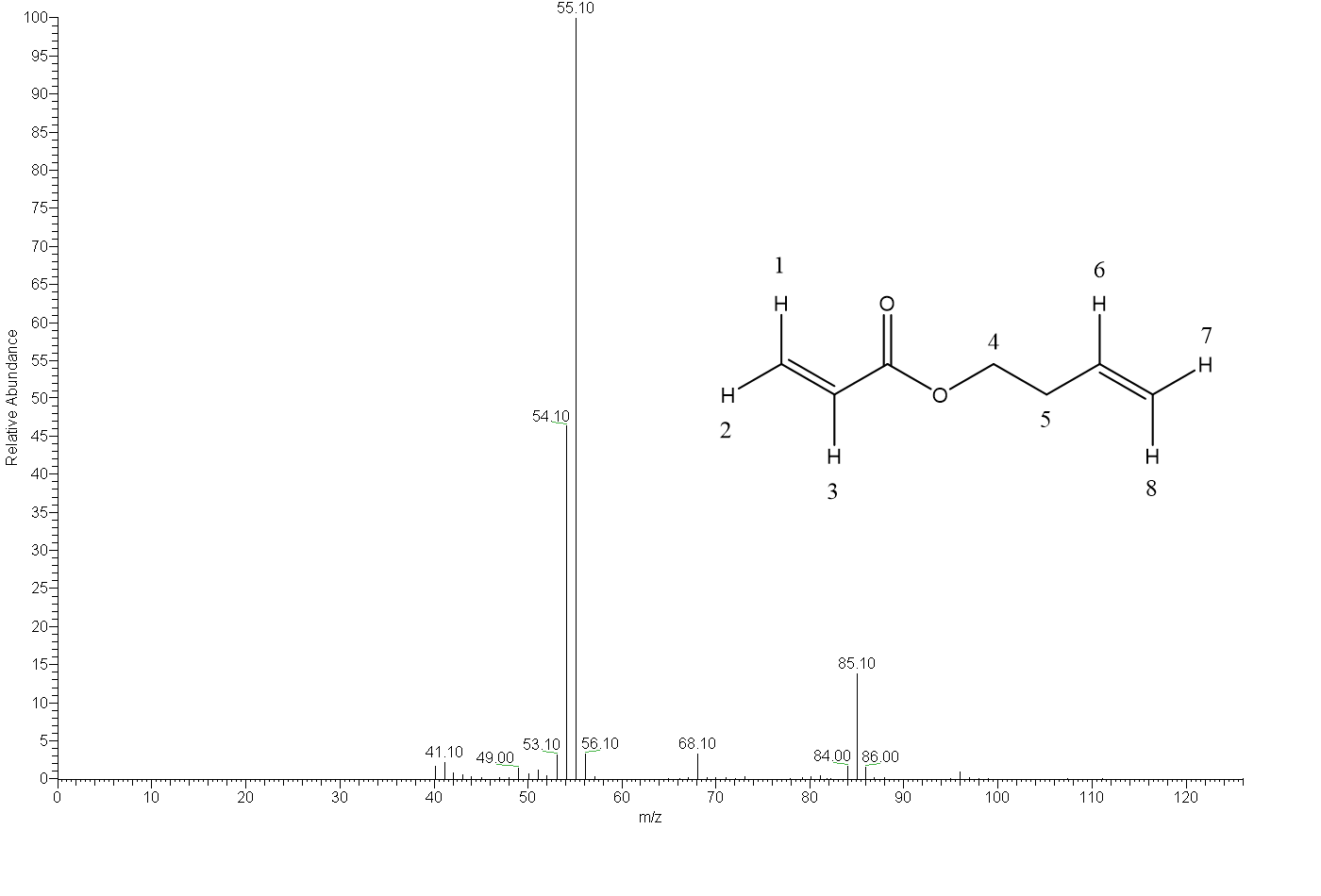


**4-Pentenyl acrylate** M = 140.18 g/mol. Yield = 437.53 mg (3.12 mmol, 52.02%), purity 98.3% (GC)

**MS-EI,** *m/z* (relative intensity in %): 55 (99.9), 68 (91.1), 67 (64.2), 41 (14.7), 53 (11.4), 69 (6.7), 40 (5.1), 73 (5.0), 56 (4.0), 84 (3.4)

**^1^H-NMR** (600 MHz, CDCl_3,_ room temperature): δ [ppm] = 6.41 (dd, J = 2.1 Hz, J’ = 17.2 Hz, 1H, **1**), 6.12 (dd, J = 12.4 Hz, J‘ = 17.5 Hz, 1H, **3**), 5.83 (m, 2H, **2** and **7**), 5.13 (d, J = 9.0 Hz, J‘ = 16.5 Hz, **8**), 4.88(d, J = 2.2 Hz, J‘ = 8.3 Hz, 1H, **9**), 4.19 (t, J = 7.1 Hz, 2H, **4**), 2.16 (q, J = 7.1 Hz,2H, **6**), 1.71 (p, J = 7.0 Hz,2H, **5**) ppm


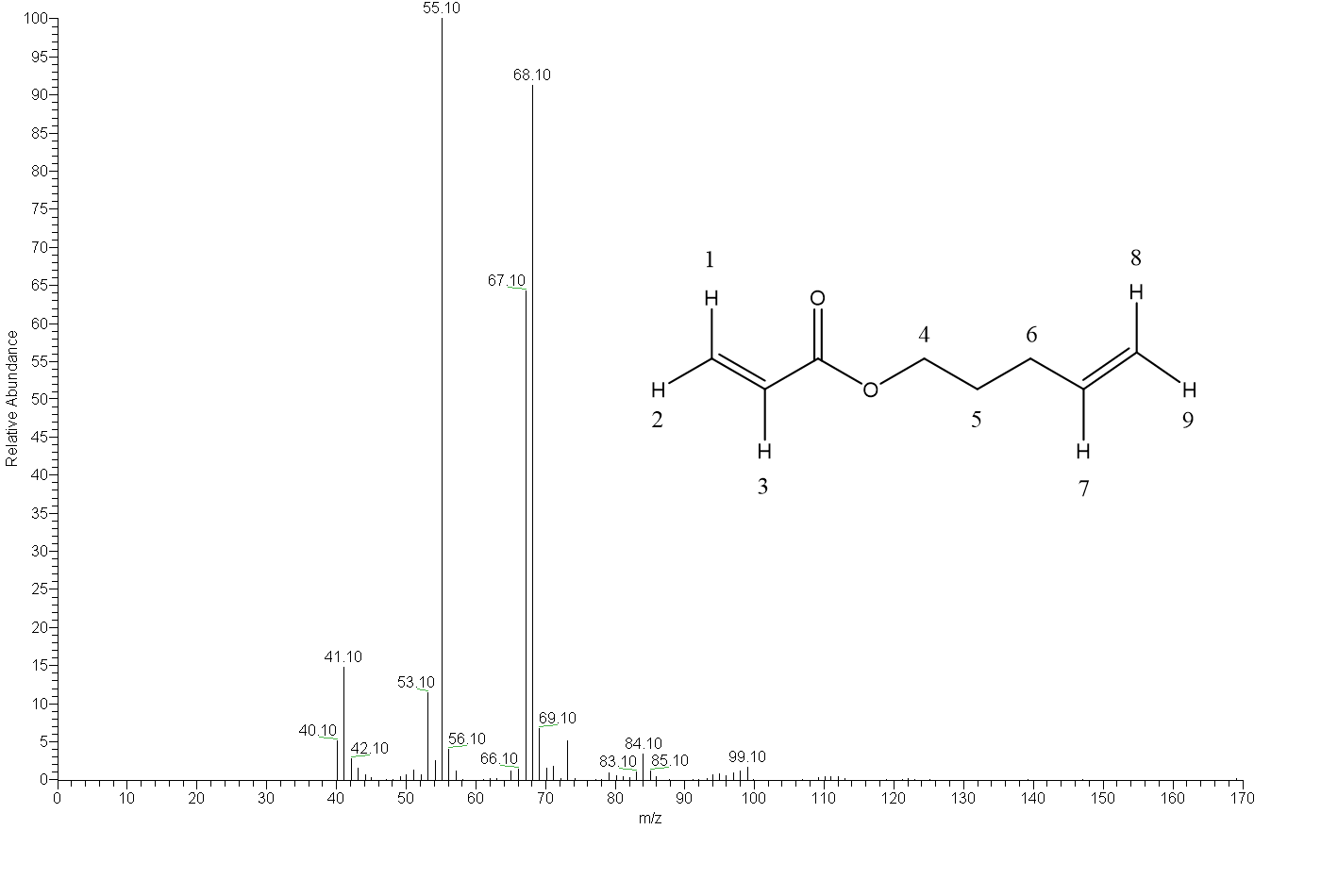


**5-Hexenyl acrylate** M = 154.21 g/mol. Yield = 481.32 mg (3.12 mmol, 52.02%), purity 99.1% (GC)

**MS-EI,** *m/z* (relative intensity in %): 55 (99.9), 54 (49.4), 67 (49.3), 82 (29.8), 41 (20.0), 81 (8.9), 73 (6.0), 53 (4.9), 84 (4.3), 56 (4.1)

**^1^H-NMR** (600 MHz, CDCl_3,_ room temperature): δ [ppm] = 6.40 (dd, J = 2.1 Hz, J’ = 17.2 Hz, 1H, **1**), 6.12 (dd, J = 12.7 Hz, J‘ = 16.8 Hz, 1H, **3**), 5.83 (m, 2H, **2** and **8**), 5.12 (d, J = 9.1 Hz, 4.87, **10**), 4.87(d, J = 8.3 Hz, 1H, **9**), 3.97 (t, J = 6.9 Hz, 2H, **4**), 2.13 (q, J = 7.1 Hz, 2H, **7**), 1.58 (m, 4H, **5** and **6**) ppm


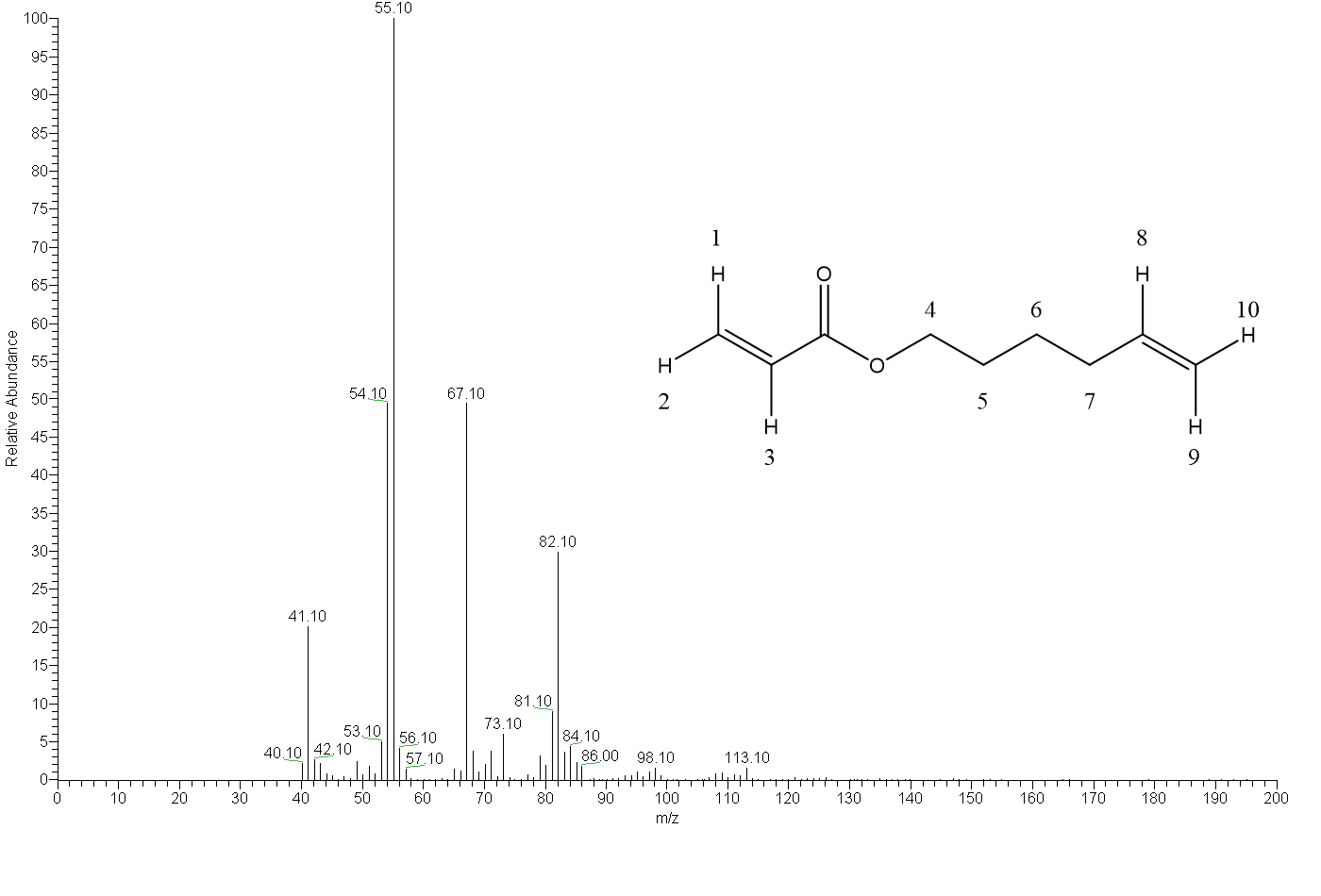


**(*E*)-2-Hexenyl acrylate** M = 154.21 g/mol. Yield = 562.37 mg (3.65 mmol, 60.78%), purity 97.7% (GC)

**MS-EI,** *m/z* (relative intensity in %): 55 (99.9), 67 (28.4), 82 (19.9), 41 (12.1), 83 (8.3), 99 (8.3), 56 (6.8), 84 (6.5), 57 (6.4), 54 (6.0)

**^1^H-NMR** (600 MHz, CDCl_3,_ room temperature): δ [ppm] = 6.41 (dd, J = 2.1 Hz, J’ = 17.2 Hz, 1H, **1**), 6.12 (dd, J = 11.7 Hz, J‘ = 16.8 Hz, 1H, **3**), 5.82 (dd, J = 2.0 Hz ,J’ = 16.9 Hz, 1H, **2**), 5.64 (m, 2H, **5** and **6**), 4.75 (d, J = 7.1 Hz, 2H, **4**), 1.98 (m, J = 7.2 Hz, 2H, **7**), 1.46 (m, J = 7.4 Hz, 2H, **8**), 0.96 (t, J = 8.0 Hz, 3H, **9**) ppm.


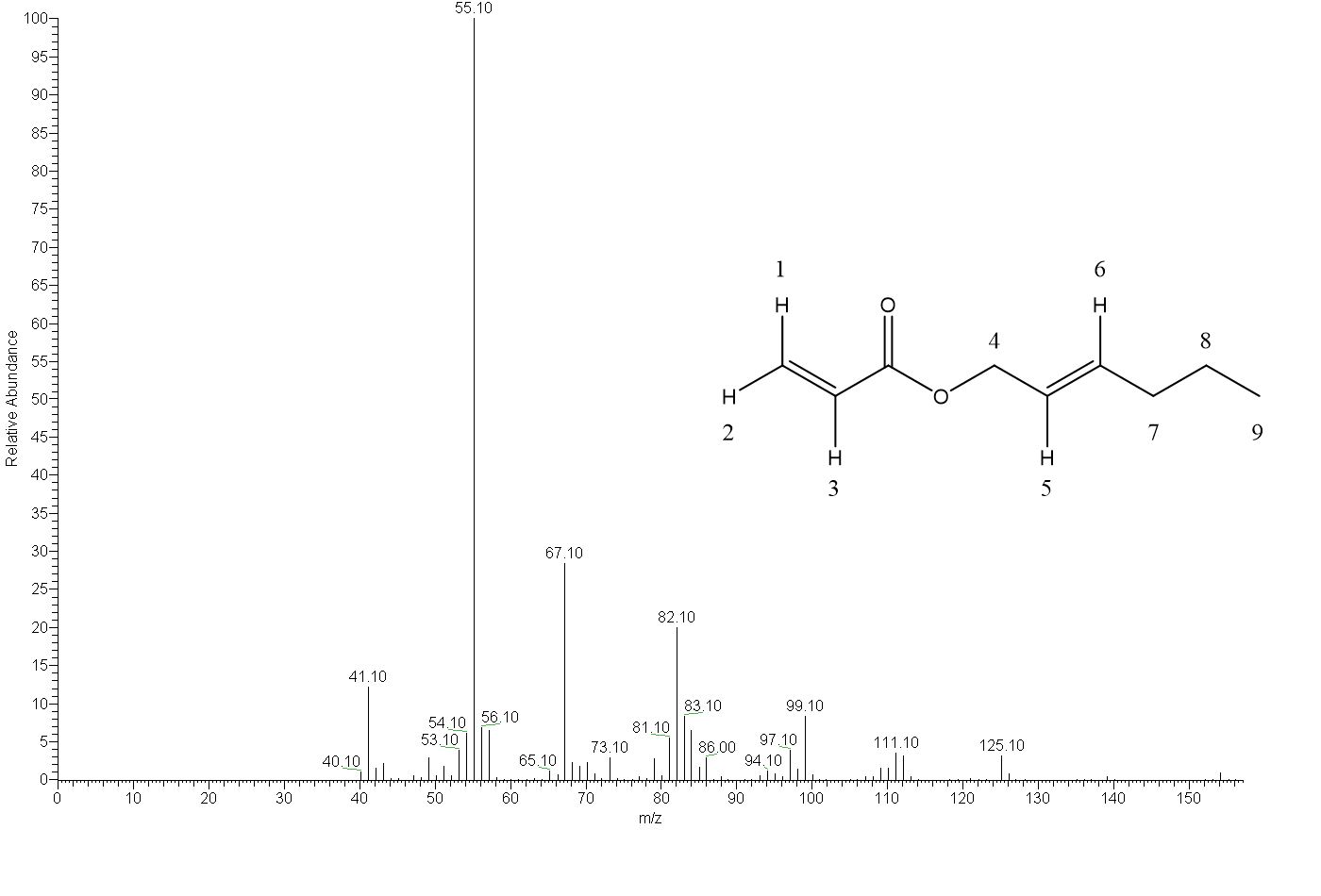


**(E)-3-Hexenyl acrylate** M = 154.21 g/mol. Yield = 572.37 mg (3,71 mmol, 61.86%), purity 98.8% (GC)

**MS-EI,** *m/z* (relative intensity in %): 67 (99.9), 55 (95.8), 82 (93.1), 41 (14.7), 81 (8.6), 54 (7.4) 83 (6.7), 68 (6.3), 53 (4.6), 56 (3.6)

**^1^H-NMR** (600 MHz, CDCl_3,_ room temperature): δ [ppm] = 6.39 (dd, J = 2.0 Hz, J’ = 17.2 Hz, 1H, **1**), 6.12 (dd, J = 11.7 Hz, J‘ = 16.8 Hz, 1H, **3**), 5.82 (dd, J = 2.0 Hz ,J’ = 16.9 Hz, 1H, **2**), 5.43 (m, 2H, **6** and **7**), 4.20 (t, J = 6.9 Hz, 2H, **4**), 2.24 (m, J = 7.2 Hz, 2H, **5**), 2.01 (m, J = 6.2 Hz, 2H, **8**), 0.79 (t, J = 8.0 Hz, 3H, **9**) ppm


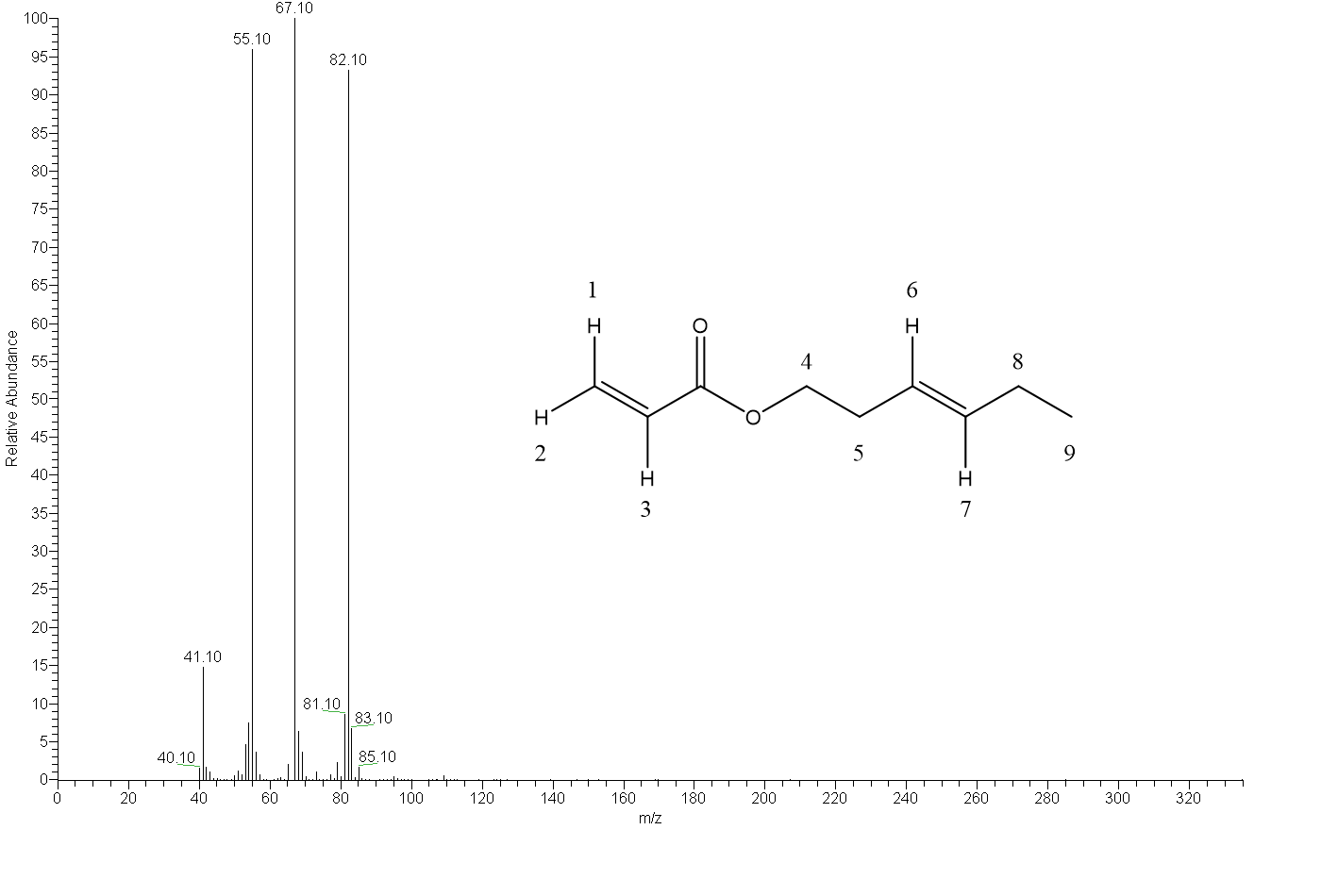


**(E)-4-Hexenyl acrylate** M = 154.21 g/mol. Yield = 560.80 mg (3.68 mmol, 60.61%), purity 96.4% (GC)

**MS-EI,** *m/z* (relative intensity in %): 67 (99.9), 82 (63.0), 55 (58.4), 81 (12.4), 54 (11.2), 41 (10.5), 68 (6.4), 53 (4.6), 83 (4.3) 79 (3.4)

**^1^H-NMR** (600 MHz, CDCl_3,_ room temperature): δ [ppm] = 6.40 (dd, J = 2.1 Hz, J’ = 17.2 Hz, 1H, **1**), 6.12 (dd, J = 12.3 Hz, J‘ = 16.8 Hz, 1H, **3**), 5.83 (dd, J = 2.0 Hz ,J’ = 16.9 Hz, 1H, **2**), 5.40 (m, 2H, **7** and **8**), 4.20 (t, J = 6.9 Hz, 2H, **4**), 2.16 (m, J = 7.1 Hz, 1H, **6**), 1.71 (p, J = 7.2 Hz, 2H, **5**), 1.63 (dd, J = 2.1 Hz, J’ = 16.9 Hz, 3H, **9**) ppm


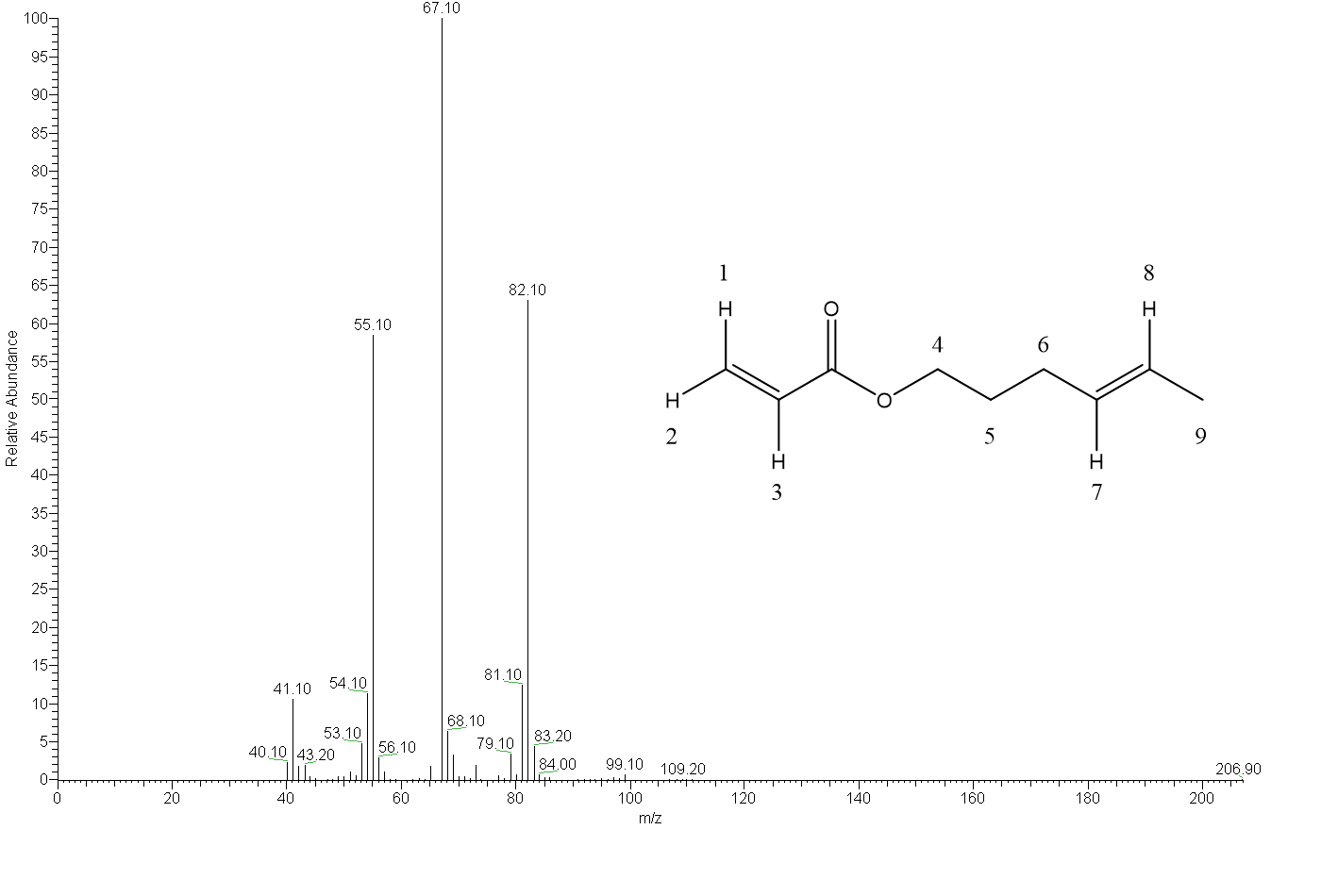


**1-Hepten-3-one** M = 112.17 g/mol. Yield = 482.3 mg (4.3 mmol, 43%), purity 97.5% (GC)

**MS-EI,** *m/z* (relative intensity in %): 55 (99.9), 70 (83.1), 97 (20.1), 41 (12.1), 83 (11.7), 57 (10.5), 56 (6.3), 42 (4.6), 85 (4.6), 71 (4.2)

**^1^H-NMR** (600 MHz, CDCl_3,_ room temperature): δ [ppm] = 6.30 (dd, J = 2.1 Hz, J’ = 17.2 Hz, 1H, **1**), 6.21 (dd, J = 12.3 Hz, J‘ = 17.1 Hz, 1H, **3**), δ = 5.91 (dd, J = 2.0 Hz, J’ = 17.1 Hz, 1H, **2**), 2.92 (t, J = 7.1Hz, 2H, **4**), 1.38-1.36 (m, 4H, **5** and **6**), 0.93 (t, J = 7.9 Hz, 3H, **1**) ppm


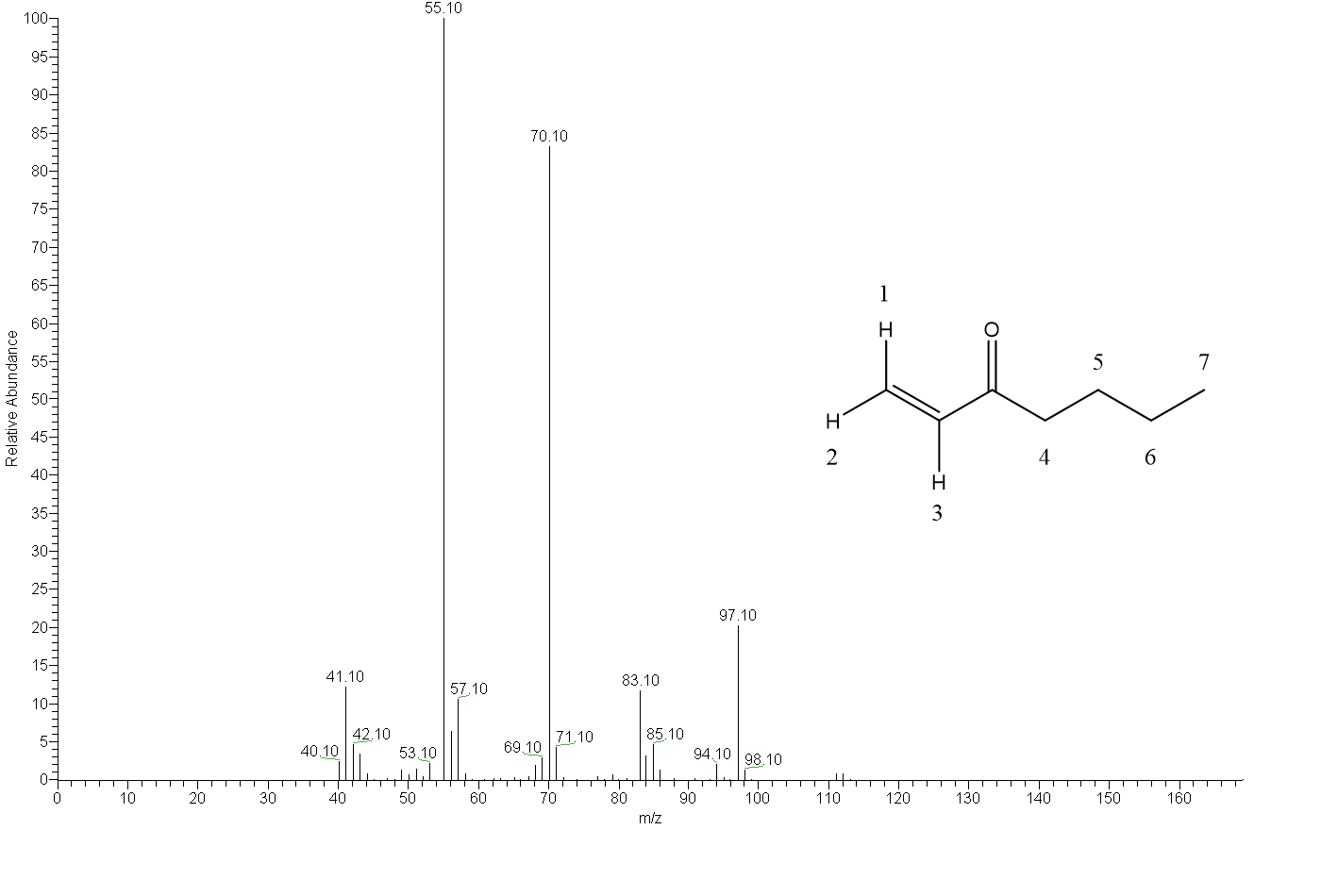


**1-Decen-3-one** M = 154.25 g/mol. Yield = 740.4 mg (4.8 mmol, 48%), purity 98.9% (GC)

**MS-EI,** *m/z* (relative intensity in %): 70 (99.9), 55 (45.7), 71 (19.0), 83 (18.9), 139 (14.5), 97 (12.2), 153 (12.0), 111 (11.6), 41 (11.0), 43 (10.5)

**^1^H-NMR** (600 MHz, CDCl_3,_ room temperature): δ [ppm] = 6.31 (dd, J = 2.0 Hz, J’ = 16.9 Hz, 1H, **3**), 6.20 (dd, J = 11.9 Hz, J‘ = 16.8 Hz, 1H, **1**), 5.91 (dd, J = 2.0 Hz, J’ = 17.0 Hz, 1H, **2**), 2,92 (t, J = 6.9 Hz, 2H, **4**), 1.36-1.23 (m, 10H, **5** - **9**), 0.88 (t, J = 7.3 Hz, 3H, **10**) ppm


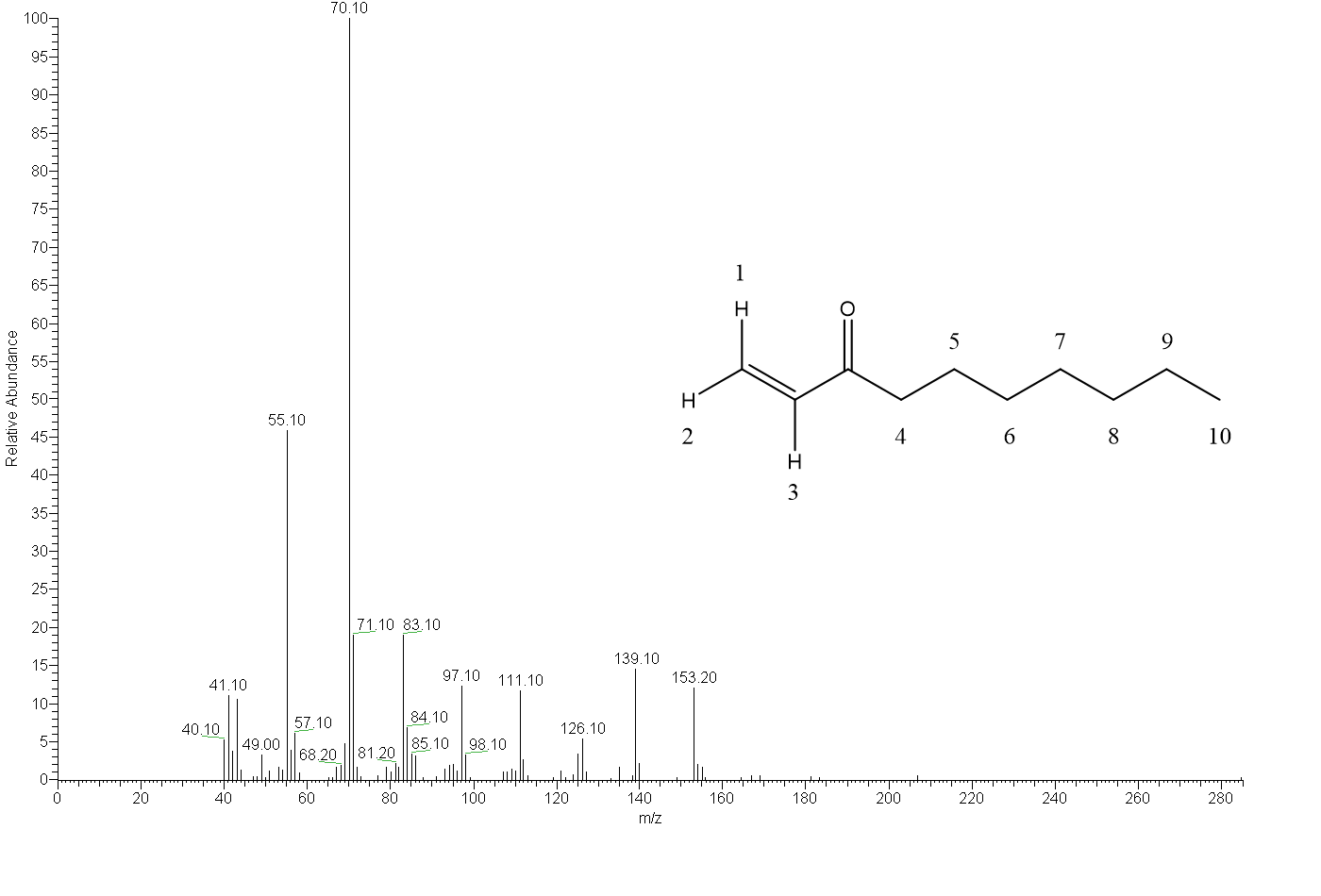


**1-Dodecen-3-one** M = 182.3 g/mol. Yield = 460 mg (2.52 mmol, 25.2%), purity 96.5% (GC)

**MS-EI,** *m/z* (relative intensity in %): 70 (99.9), 55 (47.9), 83 (18.4), 71 (17.5), 139 (15.6), 97 (12.8), 41 (12.5), 43 (12.3), 153 (12.2), 111 (11.0)

**^1^H-NMR** (600 MHz, CDCl_3,_ room temperature): δ [ppm] = 6.30 (dd, J = 2.0 Hz, J’ = 16.9 Hz, 1H, **3**), 6.21 (dd, J = 11.9 Hz, J‘ = 16.8 Hz, 1H, **1**), 5.90 (dd, J = 2.0 Hz, J’ = 16.9 Hz, 1H, **2**), 2.92 (t, J = 7.1 Hz, 2H, **4**), 1.36-1.26 (m, 14H, **5** - **11**), 0.88 (t, J = 7.1 Hz, 3H, **12**) ppm


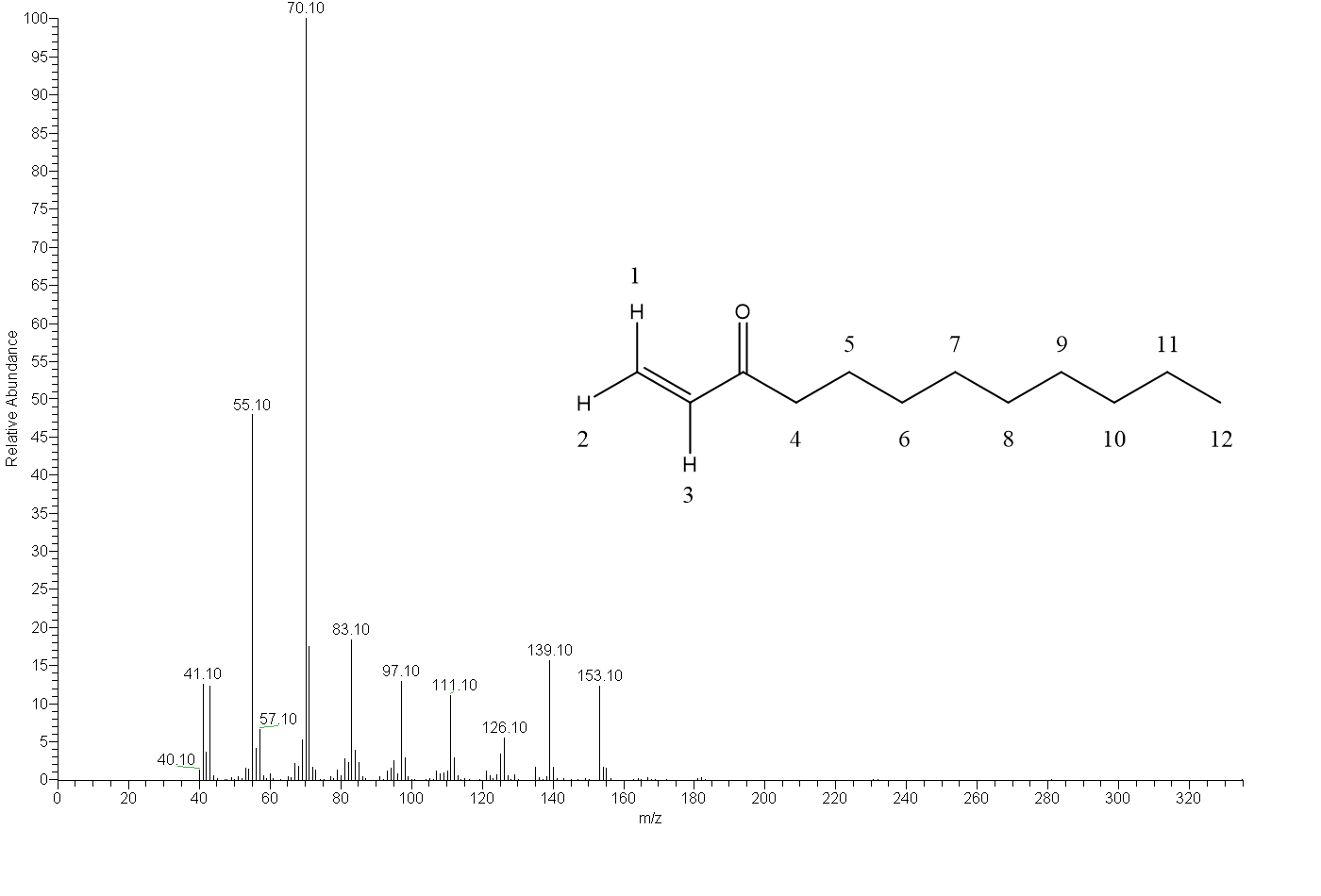


**1,7-Octadien-3-one** M = 124.18 g/mol. Yield = 187.5 mg (1.51 mmol, 20.8%), purity 96.1% (GC)

**MS-EI,** *m/z* (relative intensity in %): 41 (99.9), 67 (87.5), 55 (86.3), 54 (50.9), 82 (47.3), 93 (41.0), 80 (37.3), 79 (36.1), 83 (33.8), 57 (31.2)

**^1^H-NMR** (600 MHz, CDCl_3,_ room temperature): δ [ppm] = 6.30 (dd, J = 2.0 Hz, J’ = 16.9 Hz, 1H, **3**), 6.21 (dd, J = 11.9 Hz, J‘ = 16.8 Hz, 1H, **1**), 5.91 (dd, J = 2.0 Hz, J’ = 16.9 Hz, 1H, **2**), 5.82 (ddt J = 17.0 Hz, J’ = 10.4 Hz, J’’ = 7.6 Hz, 1H, **7**) 5.13 (d, J = 7.2 Hz, 1H, **8**), 4.88 (d, J = 6.8 Hz, 1H, **9**), 2,92 (t, J = 6.9 Hz, 2H, **4**), 2.16 (q, J = 6.9 Hz, 2H, **6**), 1.62 (p, J = 7.2 Hz, 2H, **5**)


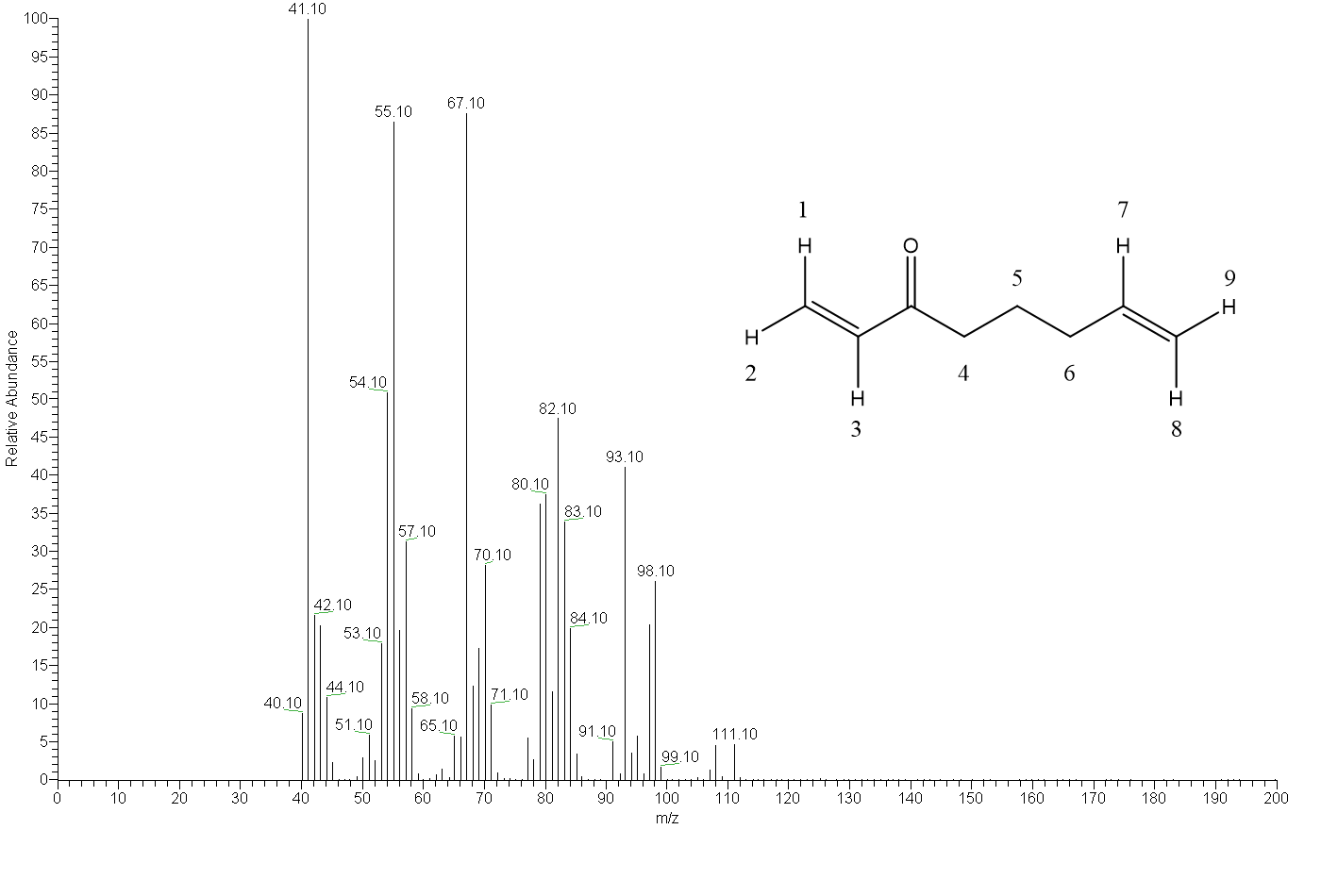


**1,8-Nonadien-3-one** M = 138.23 g/mol. Yield = 278.03 mg (2.01 mmol, 13.4%), purity 96.4% (GC)

**MS-EI,** *m/z* (relative intensity in %): 55 (99.9) 70 (56.5), 83 (29.5), 96 (22.9), 41 (16.4), 68 (16.2), 97 (15.0), 67 (12.1), 109 (11.8), 95 (11.4)

**^1^H-NMR** (600 MHz, CDCl_3,_ room temperature): δ [ppm] = 6.30 (dd, J = 2.0 Hz, J’ = 17.1 Hz, 1H, **3**), 6.22 (dd, J = 11.9 Hz, J‘ = 16.8 Hz, 1H, **1**), 5.91 (dd, J = 2.0 Hz, J’ = 17.3 Hz, 1H, **2**), 5.82 (ddt, J = 17.0 Hz, J’ = 10.4 Hz, J’’ = 7.5 Hz, 1H, **8**), 5.13 (d, J = 8.7 Hz 1H, **9**), 4.88 (d, J = 9.1 Hz, 1H, **10**), 2,92 (t, J = 6.9 Hz, 2H, **4**), 2.13 (q, J = 7.1 Hz, 2H, **4**), 1.34 (p, J = 7.4 Hz, 2H, **6**), 1.26 (p, J = 7.4 Hz, 2H, **5**), ppm


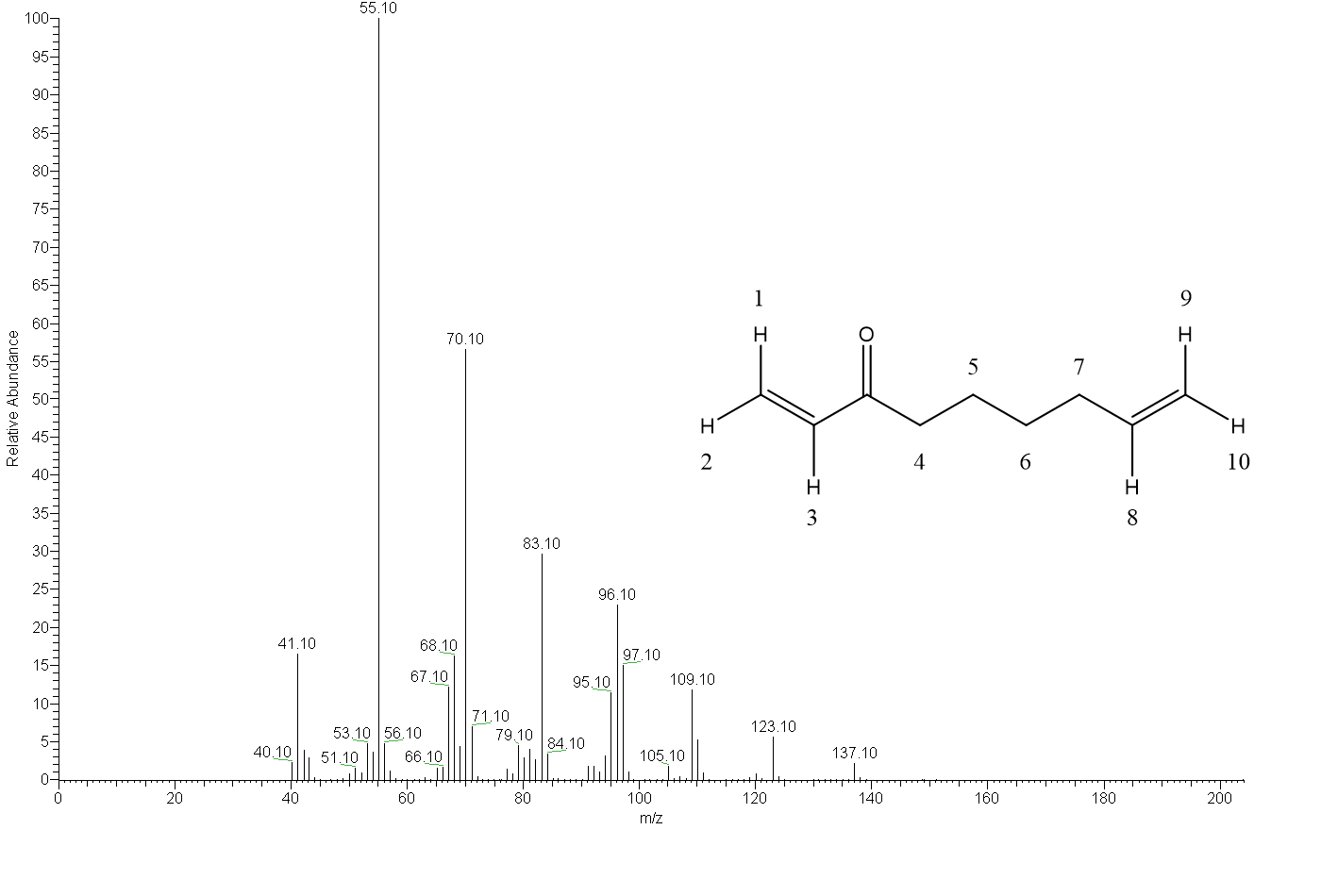


**1,9-Decadien-3-one** M = 152.23 g/mol. Yield = 246.47 mg (1.62 mmol, 10,8%), purity 95.9% (GC)

**MS-EI,** *m/z* (relative intensity in %): 55 (99.9), 70 (70.1), 109 (24.5), 83 (22.1), 41 (15.8), 67 (10.9), 95 (10.8), 96 (9.8), 97 (9.7), 81 (8.5)

**^1^H-NMR** (600 MHz, CDCl_3,_ room temperature): δ [ppm] = 6.31 (dd, J = 2.0 Hz, J’ = 16.9 Hz, 1H, **3**), 6.19 (dd, J = 12.1 Hz, J‘ = 16.8 Hz, 1H, **1**), 5.91 (dd, J = 2.0 Hz, J’ = 16.9 Hz, 1H, **2**), 5.82 (ddt, , J = 17.0 Hz, J’ = 10.4 Hz, J’’ = 7.4 Hz, 1H, **9**), 5.12 (d, J = 8.7 Hz 1H, **11**), 4.87 (d, J = 9.1 Hz, 1H, **10**), 2.92 (t, J = 6.9 Hz, 2H, **4**), 2.13 (q, J = 6.2 Hz, 2H, **8**), 1.38-1.29 (m, 6H, **5** - **7**) ppm


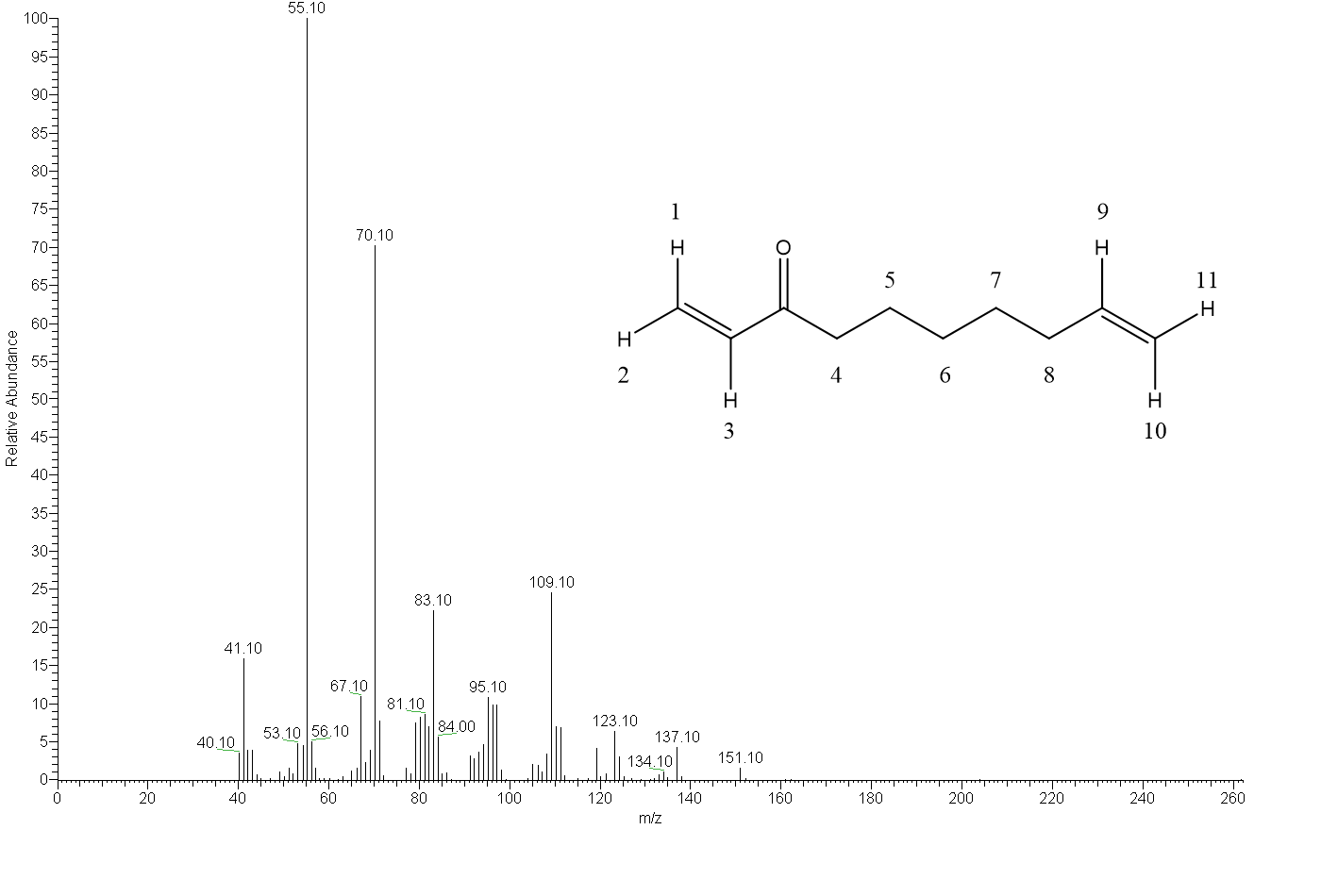


**(*E*)-1,6-Decadien-3-one** M = 152.23 g/mol. Yield = 261.60 mg (1.72 mmol, 5.1%), purity 96.4% (GC)

**MS-EI,** *m/z* (relative intensity in %): 55 (99.9), 109 (38.5), 82 (36.6), 67 (36.4), 95 (25.3), 81 (17.8), 96 (17.4), 41 (15.2), 83 (11.2), 70 (10.9)

**^1^H-NMR** (600 MHz, CDCl_3,_ room temperature): δ [ppm] = 6.30 (dd, J = 2.1 Hz, J’ = 17.1 Hz, 1H, **3**), 6.21 (dd, J = 11.9 Hz, J‘ = 16.8 Hz, 1H, **1**), 5.91 (dd, J = 2.0 Hz, J’ = 16.9 Hz, 1H, **2**), 5.43-5.42 (m /overlap of two dt, 2H, **6** and **7**), 2.92 (t, J = 7.1 Hz, 2H, **4**), 2.07 (m, J = 6.9 Hz, 2H, **5**), 1.98 (m, J = 6.9 Hz, 2H, **8**), 1.46 (sextet, J = 7.2 Hz, 2H, **9**), 0.96 (t, J = 7.1 Hz, 3H, **10**)


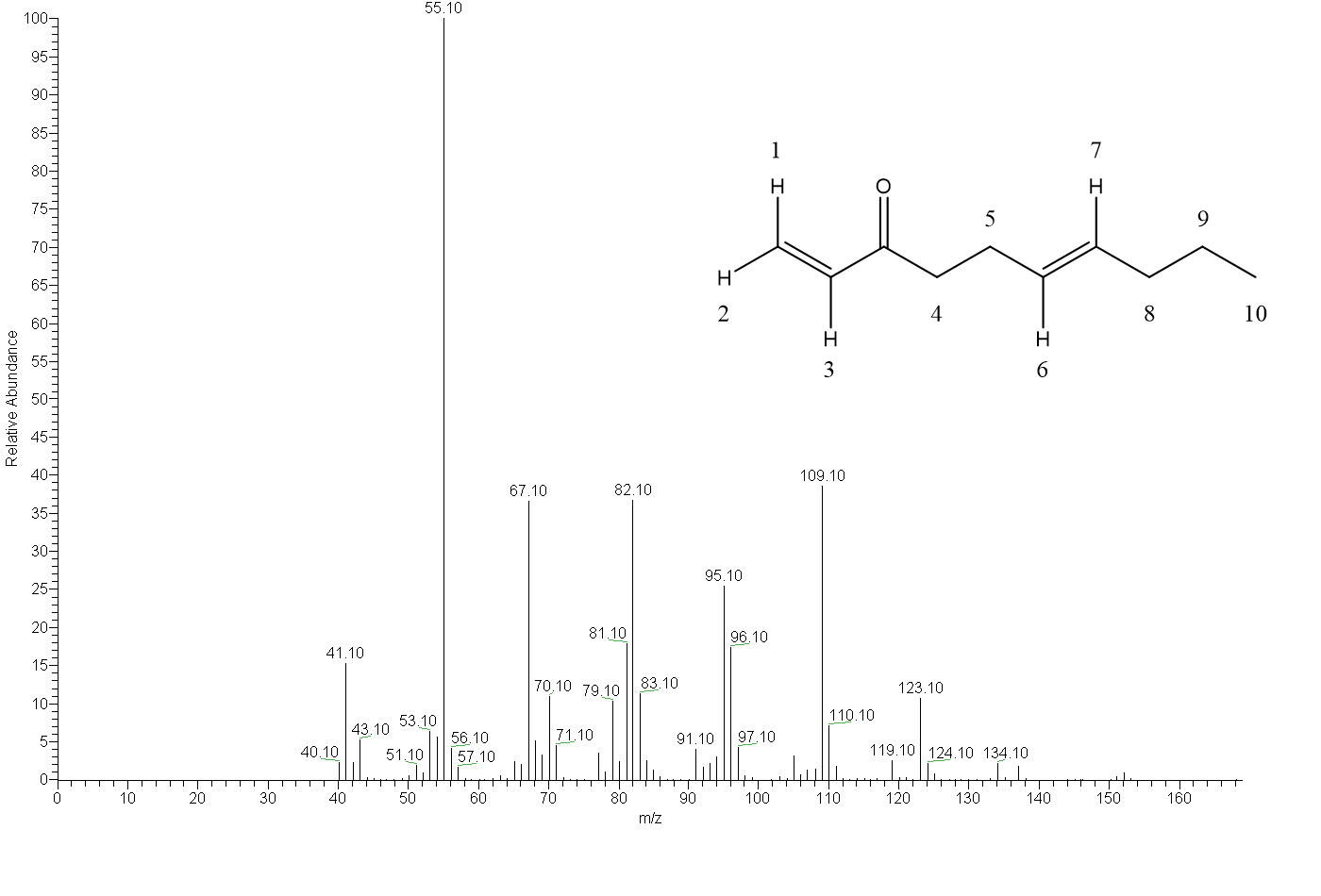


**(*E*)-1,7-Decadien-3-one** M = 152.23 g/mol. Yield = 221.3 mg (1.45 mmol, 2.91%), purity 96.9% (GC)

**MS-EI,** *m/z* (relative intensity in %): 55 (99.9), 70 (71.7), 109 (24.8), 83 (22.4), 41 (16.3), 95 (11.2), 67 (11.0), 81 (10.4), 96 (9.7), 97 (9.5)

**^1^H-NMR** (600 MHz, CDCl_3,_ room temperature): δ [ppm] = 6.31 (dd, J = 2.1 Hz, J’ = 17.1 Hz, 1H, **3**), 6.21 (dd, J = 12.1 Hz, J‘ = 16.9 Hz, 1H, **1**), 5.91 (dd, J = 2.0 Hz, J’ = 16.9 Hz, 1H, **2**), 5.43-5.41 (m / overlap of two dt, 2H, **7** and **8**), 2.92 (t, J = 6.9 Hz, 2H, **4**), 2.16 (m, J = 6.9 Hz, 2H, **6**), 2.00 (m, J = 6.9 Hz, 2H, **9**), 1.62 (p, J = 7.2 Hz, 2H, **5**), 0.79 (t, J = 7.1 Hz, 3H, **10**) ppm


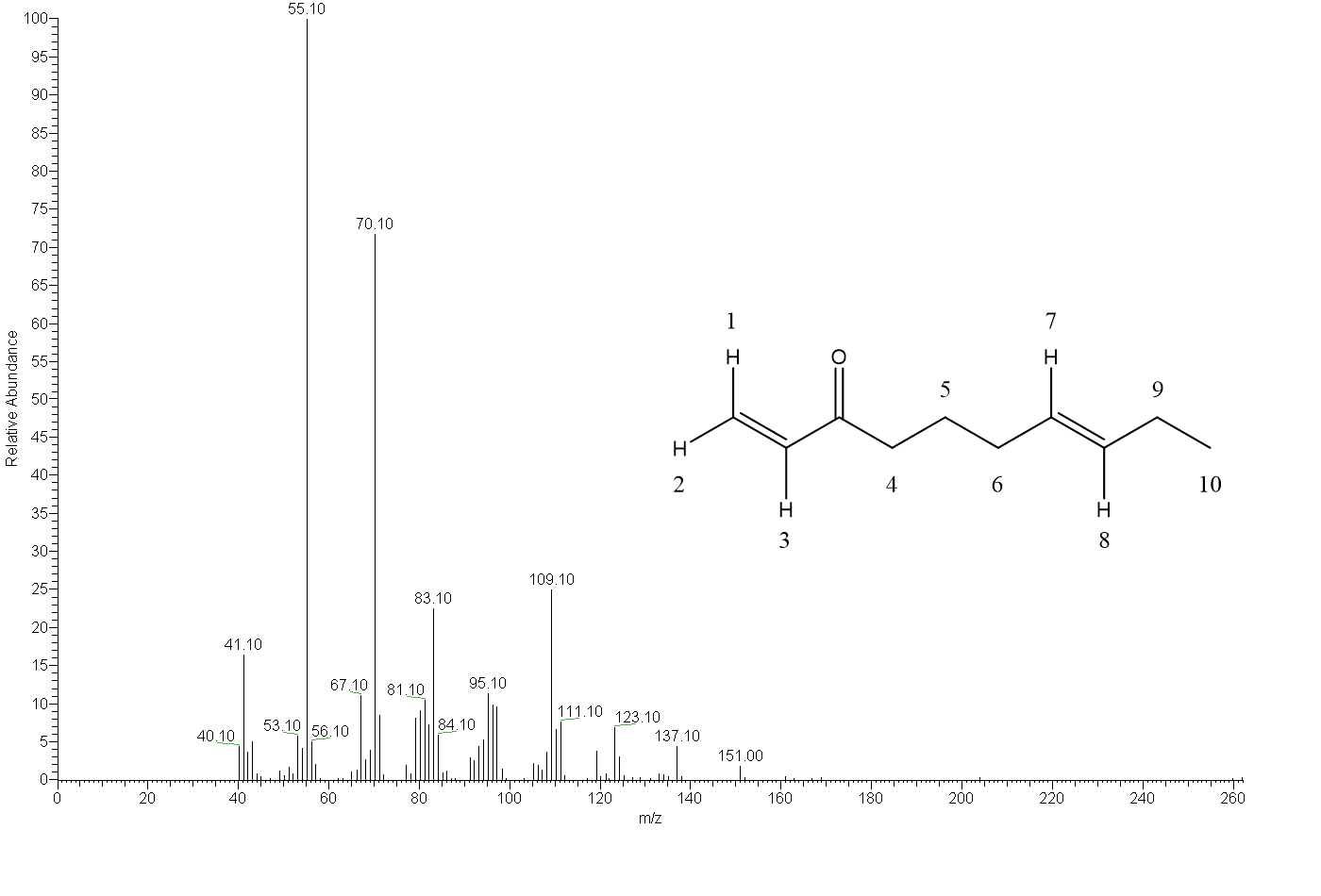


**(*E*)-1,8-Decadien-3-one** M = 152.23 g/mol. Yield = 243.3 mg (1.59 mmol, 6.57%), purity 97.3% (GC)

**MS-EI,** *m/z* (relative intensity in %): 55 (99.9), 83 (43.3), 67 (41.6), 97 (31.4), 70 (30.8), 81 (23.8), 82 (21.1), 41 (16.9), 95 (14.3), 96 (13.0)

**^1^H-NMR** (600 MHz, CDCl_3,_ room temperature): δ [ppm] = 6.30 (dd, J = 2.0 Hz, J’ = 16.9 Hz, 1H, **3**), 6.20 (dd, J = 11.9 Hz, J‘ = 16.8 Hz, 1H, **1**), 5.91 (dd, J = 2.0 Hz, J’ = 16.9 Hz, 1H, **2**), 5.43-5.41 (m / overlap of two dt, 2H, **8** and **9**), 2.92 (t, J = 6.9 Hz, 2H, **4**), 2.16 (m, J = 7.1 Hz, 2H, **7**) 1.63 (m, J = 7.2 Hz, 3H, **10**), 1.36-1.29 (m / overlap of two p, 4H, **5** and **6**) ppm


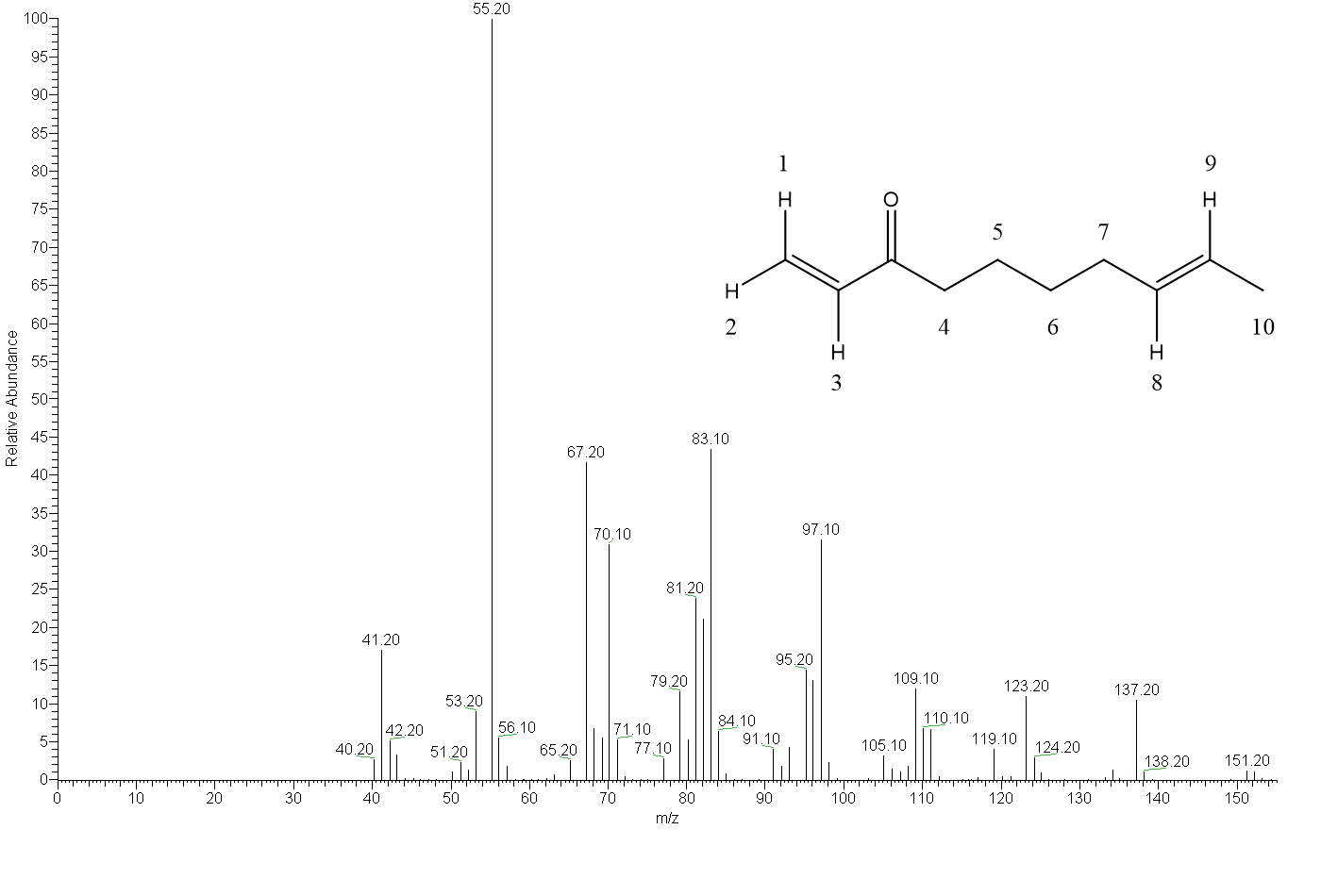

 Figure 1: Reaction pathways used for the synthesis of the 1-alken-3-ones and their unsaturated derivatives. Substance no. 24 was synthesized using pathway a). Substance no. 26 was synthesized using pathway b) and substance no. 25 via pathway c). Substance no. 22 was obtained using pathway d). Substances no. 20, 21 and 23 were synthesized using the corresponding alcohol following pathway e). Following pathway f) substances 15 and 18 could be obtained. All numbers are according to table 1 in the manuscript.

Figure 2: Synthesis route for acrylate derivatives

Table 1: Individual odour thresholds of acrylates determined by each panellists via gas chromatography olfactometry using (*E*)-2-decenal as internal standard

| no. | substance | odour threshold [ng/l_air_]^a^ | | | | | | | | |
| --- | --- | --- | --- | --- | --- | --- | --- | --- | --- | --- |
|  |  | P 1 | P 2 | P 3 | P 4 | P 5 | P6 | P7 | mean odour threshold | median odour threshold |
| 1 | ethyl acrylate | 0.39 | 0.78 | 0.20 | 0.20 | 0.20 |  |  | 0.30 | 0.20 |
| 2 | propyl acrylate | 0.98 | 1.96 | 0.49 | 1.96 | 0.12 |  |  | 0.74 | 0.98 |
| 3 | butyl acrylate | 15.17 | 7.58 | 1.90 | 15.17 | 1.90 | 0.47 | 5.69 | 6.84 | 5.69 |
| 4 | pentyl acrylate | 1.94 | 1.94 | 0.48 | 7.74 | 0.12 |  |  | 1.11 | 1.94 |
| 5 | hexyl acrylate | 5.73 | 11.46 | 2.86 | 2.86 | 1.43 |  |  | 3.78 | 2.86 |
| 6 | heptyl acrylate | 8.98 | 4.49 | 0.56 | 2.25 | 1.12 |  |  | 2.25 | 2.25 |
| *7* | octyl acrylate | 2.81 | 2.81 | 11.23 | 1.40 | 1.40 |  |  | 2.81 | 2.81 |
| 8 | 3-butenyl acrylate | 7.41 | 7.41 | 0.93 | 14.82 | 0.23 |  |  | 2.81 | 7.41 |
| 9 | 4-pentenyl acrylate | 1.78 | 0.44 | 0.44 | 0.11 | 0.22 |  |  | 0.39 | 0.44 |
| 10 | 5-hexenyl acrylate | 0.70 | 2.81 | 5.62 | 2.81 | 0.35 | 0.18 | 22.46 | 1.71 | 2.81 |
| 11 | (E)-4-hexenyl acrylate | 0.09 | 0.09 | 0.02 | 0.04 | 0.04 | 0.02 | 0.18 | 0.05 | 0.04 |
| 12 | (E)-3-hexenyl acrylate | 0.01 | 0.03 | 0.003 | 0.01 | 0.003 |  |  | 0.01 | 0.01 |
| 13 | (E)-2-hexenyl acrylate | 3.96 | 0.99 | 0.49 | 7.91 | 0.99 |  |  | 1.72 | 0.99 |
| 14 | 1-hexen-3-one | 0.003 | 0.01 | 0.05 | 0.003 | 0.003 | 0.002 | 0.01 | 0.01 | 0.003 |
| 15 | 1-hepten-3-one | 0.03 | 0.05 | 0.21 | 0.11 | 0.01 | 0.01 | 0.11 | 0.05 | 0.05 |
| 16 | 1-octen-3-one | 0.01 | 0.01 | 0.12 | 0.06 | 0.004 | 0.03 | 0.23 | 0.03 | 0.03 |
| 17 | 1-nonen-3-one | 0.004 | 0.01 | 0.12 | 0.03 | 0.01 | 0.03 | 0.12 | 0.03 | 0.03 |
| 18 | 1-decen-3-one | 0.18 | 0.72 | 5.76 | 0.72 | 0.36 | 0.72 | 11.52 | 1.07 | 0.72 |
| 19 | 1-undencen-3-one | 1.91 | 7.64 | 1.91 | 0.48 | 1.91 | 0.96 | 244.5 | 3.46 | 1.91 |
| 20 | 1-dodecen-3-one | 13.74 | 54.95 | 109.9 | 109.9 | 13.74 | 27.47 | 219.8 | 49.77 | 54.95 |
| 21 | 1,7-octadien-3-one | 1.83 | 0.92 | 1.83 | 0.23 | 0.06 | 0.92 | 0.91 | 0.62 | 0.92 |
| 22 | 1,8-nonadien-3-one | 0.01 | 0.12 | 0.03 | 3.77 | 0.03 | 0.12 | 7.54 | 0.18 | 0.12 |
| 23 | 1,9-decadien-3-one | 0.11 | 0.46 | 0.11 | 0.23 | 0.11 | 3.66 | 0.92 | 0.34 | 0.23 |
| 24 | (E)-1,8-decadien-3-one | 3.92 | 7.84 | 3.92 | 15.67 | 7.84 | 15.67 | 31.34 | 9.55 | 7.84 |
| 25 | (E)-1,7-decadien-3-one | 5.58 | 5.58 | 5.58 | 2.79 | 11.17 | 22.33 | 11.17 | 7.51 | 5.58 |
| 26 | (E)-1,6-decadien-3-one | 0.74 | 0.05 | 0.01 | 0.37 | 0.74 |  |  | 0.16 | 0.37 |

^a^ Odour thresholds were determined as described by Ullrich and Grosch [6]

References

1. Xiao Q, He Q, Li J, Wang J. 1,4-Diazabicyclo[2.2.2]octane-Promoted Aminotrifluoromethylthiolation of alpha,beta-Unsaturated Carbonyl Compounds: N-Trifluoromethylthio-4-nitrophthalimide Acts as Both the Nitrogen and SCF3 Sources. Org Lett. 2015;17(24):6090-3. doi:10.1021/acs.orglett.5b03116.

2. van den Nieuwendijk Adrianus MCH, Kriek Nicole MAJ, Brussee J, van Boom Jacques H, van der Gen A. Stereoselective Synthesis of (2R,5R)- and (2S,5R)-5-Hydroxylysine. Eur J Org Chem. 2000;2000(22):3683-91. doi:10.1002/1099-0690(200011)2000:22<3683::aid-ejoc3683>3.0.co;2-u.

3. Vakhidov RR, Alekseev SB. Synthesis of 5Z,9E-tridecadien-1-ylacetate, an attractant of Trichoplusia ni. Chemistry of Natural Compounds. 2011;47(1):94-5. doi:10.1007/s10600-011-9838-z.

4. Brown DC, Nichols SA, Gilpin AB, Thompson DW. Transition metal promoted alkylations of unsaturated alcohols. Alkylation of alkynols with organoalanes promoted by Group IVA metal-cyclopentadienyl compounds. J Org Chem. 1979;44(20):3457-61. doi:10.1021/jo01334a002.

5. Hara S, Kishimura K, Suzuki A. Reaction of organoboranes with the dianion of phenoxyacetic acid. The first direct synthesis of carboxylic acids from organoboranes. Tetrahedron Letters. 1978;19(32):2891-994. doi:10.1016/s0040-4039(01)94891-9.

6. Ullrich F, Grosch W. Identification of the most intense volatile flavour compounds formed during autoxidation of linoleic acid. Zeitschrift für Lebensmittel-Untersuchung und Forschung. 1987;184(4):277-82.
